# Tick-to-host transmission differs between *Borrelia afzelii* strains

**DOI:** 10.1128/spectrum.01675-23

**Published:** 2023-09-07

**Authors:** Dolores Genné, Whitney Jiricka, Anouk Sarr, Maarten J. Voordouw

**Affiliations:** 1 Laboratory of Ecology and Evolution of Parasites, Institute of Biology, University of Neuchâtel, Neuchâtel, Switzerland; 2 Department of Veterinary Microbiology, Western College of Veterinary Medicine, University of Saskatchewan, Saskatoon, Saskatchewan, Canada; Uniwersytet Medyczny w Bialymstoku, Bialystok, Poland

**Keywords:** *Borrelia afzelii*, *Ixodes ricinus*, Lyme borreliosis, mixed infection, multiple-strain infection, pathogen strain, tick, tick-borne pathogens, transmission, vector-borne pathogen

## Abstract

Many vector-borne pathogens establish multiple-strain infections in the vertebrate host and the arthropod vector. Multiple-strain infections in the host influence strain acquisition by naive vectors. Whether multiple-strain infections in the vector influence strain-specific transmission to naive hosts remains unknown. The spirochete, *Borrelia afzelii*, causes Lyme borreliosis and multiple-strain infections are common in both the tick vector and vertebrate host. Our study used two *B. afzelii* strains: Fin-Jyv-A3 and NE4049. Donor mice were infected with Fin-Jyv-A3 alone, NE4049 alone, or with both strains. Larval ticks fed on donor mice and molted into nymphal ticks infected with either strain or both strains. These nymphs were fed on test mice to determine whether multiple-strain infections in the nymph influence nymph-to-host transmission (NHT). Multiple-strain infection in the donor mice reduced the acquisition of both strains by ticks by 23%. Thus, a substantial fraction of infected nymphs from the multiple strain treatment were infected with the “wrong” competitor strain rather than the “right” focal strain. As a result, nymphs from the multiple strain treatment were 46% less likely to infect the test mice with the focal strain compared to nymphs from the single strain treatment. However, multiple-strain infection in the nymphal tick had no effect on the NHT of either strain. The nymphal spirochete load of Fin-Jyv-A3 was 1.9 times higher compared to NE4049. NHT of Fin-Jyv-A3 (79%) was 1.5 times higher compared to NE4049 (53%). Our study suggests that *B. afzelii* strains with higher nymphal spirochete loads have higher NHT.

**IMPORTANCE:**

For many vector-borne pathogens, multiple-strain infections in the vertebrate host or arthropod vector are common. Multiple-strain infections in the host reduce strain acquisition by feeding vectors. Whether multiple-strain infections in the vector influence strain transmission to the host remains unknown. In our study, we used two strains of the tick-borne spirochete *Borrelia afzelii*, which causes Lyme borreliosis, to investigate whether multiple-strain infections in the nymphal tick influenced nymph-to-host transmission (NHT) of strains. Multiple-strain infections in mice reduced the acquisition of both *B. afzelii* strains by nymphal ticks. As a result, nymphs from the multiple strain treatment were less likely to infect naive test mice with the focal strain. Multiple-strain infection in the nymphal ticks did not influence the NHT of either strain. The strain with the higher bacterial abundance in the nymph had higher NHT. Our study suggests that pathogen abundance in the arthropod vector is important for vector-to-host transmission.

## INTRODUCTION

Many genetically diverse pathogens consist of multiple strains. Multiple-strain infections or mixed infections occur when the host is infected with two or more strains belonging to the same pathogen species (1 – 5). As closely related pathogen strains occupy the same ecological niche, multiple-strain infections are likely to lead to competitive interactions between strains over limited host resources (6 – 11). Closely related pathogen strains can also be targeted by the same host immune responses, which is referred to as apparent competition (4, 12 – 14). Regardless of their source, competitive interactions can reduce the presence and/or abundance of strains in host tissues, which in turn can reduce strain transmission to new hosts (6 – 11). Thus, multiple-strain infections can have important consequences for the epidemiology of genetically diverse pathogens.

Vector-borne pathogens require both an arthropod vector and a vertebrate host to complete their life cycle. The two critical steps in the life cycle of any vector-borne pathogen are the following: (i) pathogen acquisition by naive vectors feeding on infected hosts (also referred to as host-to-vector transmission) and (ii) transmission from infected vectors to naive hosts (vector-to-host transmission). For vector-borne pathogens, infections with multiple strains occur in both the vertebrate host and the arthropod vector (1, 2, 15 – 18). Multiple-strain infections in the vertebrate host reduce strain acquisition by naive vectors (6, 19 – 22). Whether this reduced strain acquisition by the vector results in reduced vector-to-host transmission remains unknown. Studies on multiple-strain infections in arthropod vectors have found evidence for competition (19, 23) and facilitation (17) with respect to strain abundance in the vector. Whether multiple-strain infections in the arthropod vector influence vector-to-host transmission of the constituent strains remains unknown.

We use the tick-borne pathogen *Borrelia afzelii* to study how interactions between strains influence their acquisition by ticks and their transmission to hosts. This spirochete bacterium belongs to the *B. burgdorferi sensu lato* (sl) genospecies complex and is one of the most common causes of Lyme borreliosis in Europe (24). *Borrelia afzelii* is transmitted by *Ixodes ricinus* ticks and uses small mammals as reservoir hosts (24). The life cycle of *I. ricinus* consists of three post-egg stages: larva, nymph, and adult. Transovarial transmission of *B. burgdorferi* sl is rare (25, 26); *I. ricinus* larvae acquire the bacterium after feeding on infected hosts. Newly infected larvae molt into infected nymphs, which overwinter and transmit the bacterium back to the vertebrate host population in the following year. Adult female ticks require a blood meal to produce eggs, and they usually feed on larger mammals (e.g., deer), which are incompetent hosts for *B. afzelii* (27, 28). The nymph is, therefore, the most important stage for the transmission of *B. afzelii*, and the density of infected nymphs determines the risk of Lyme borreliosis (29).

Populations of *B. afzelii* consist of multiple genetically distinct strains (8, 30). In areas where *B. afzelii* is endemic, infections with multiple strains are common in both the vertebrate host (8, 14) and the *I. ricinus* tick vector (30, 31). A field survey found an association between the frequency of a strain in the *B. afzelii* population and its bacterial abundance in nymphs (31). This observation suggests that variation among strains in bacterial abundance in nymphs is associated with variation in nymph-to-host transmission success. *Ixodes ricinus* nymphs that fed as larvae on mice infected with two strains of *B. afzelii* have reduced acquisition of each strain compared to nymphs that fed as larvae on mice infected with a single strain (19, 23). In the present study, we used nymphs experimentally infected with single strains or infected with two strains to investigate three questions. First, does the reduced acquisition of strains by *I. ricinus* nymphs that fed as larvae on multiple-strain infected mice result in reduced nymph-to-host transmission in the next step of the life cycle? Second, do *B. afzelii* strains differ in their probability of nymph-to-host transmission? Third, does multiple-strain infection in the nymph reduce the probability of nymph-to-host transmission for the constituent strains?

## MATERIALS AND METHODS

### Mice, ticks, and *Borrelia afzelii* strains

Hundred female, 5-week-old, pathogen-free *Mus musculus* BALB/c mice were used in this study. *Ixodes ricinus* ticks were obtained from a specific pathogen-free laboratory colony that has been maintained since 1978 at the University of Neuchâtel (32). The two strains of *B. afzelii* used in this study are Fin-Jyv-A3 and NE4049, which were isolated from a bank vole (*Myodes glareolus*) in Finland and an *I. ricinus* tick in Switzerland, respectively. These two strains exist in the *Borrelia* multi-locus sequence type (MLST) database under strain ID numbers 1961 and 1887. Both strains have been genotyped with respect to their *ospC* (outer surface protein C) major group (oMG) and their MLST. Strain Fin-Jyv-A3 has oMG A3 and MLST 676, whereas strain NE4049 has oMG A10 and MLST 679.

### Creation of the challenge nymphs

**Fig 1:**
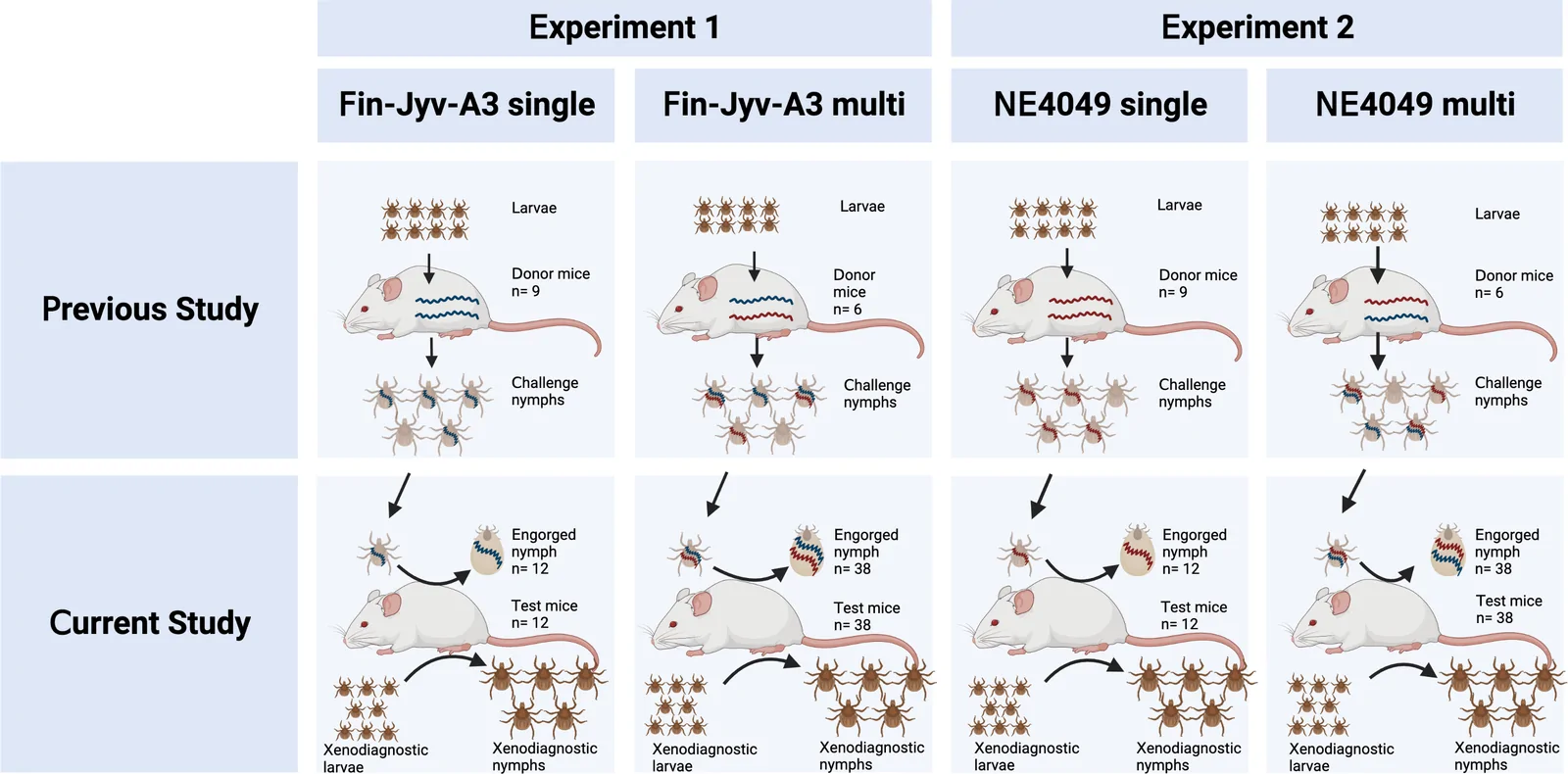
This study was divided into two independent experiments. In experiment 1 (columns 1 and 2), the focal strain was Fin-Jyv-A3, whereas in experiment 2 (columns 3 and 4), the focal strain was NE4049. Each experiment included a single-strain infection treatment (columns 1 and 3) and a multiple-strain infection treatment (columns 2 and 4). In a previous study (top row), we fed larvae on the experimentally infected donor mice and the engorged larvae molted into the challenge nymphs. For each donor mouse, we tested a random sample of 1-month-old and 4-month-old unfed challenge nymphs to determine the acquisition of the focal strain. In the current study (bottom row), we infested each of the 100 test mice with a single challenge nymph. Of the 100 challenge nymphs, 12, 38, 12, and 38 came from donor mice infected with Fin-Jyv-A3 alone, infected with both strains, infected with strain NE4049 alone, and infected with both strains. The resultant engorged challenge nymphs were tested to determine their strain-specific infection status. The strain-specific infection status of the test mice was based on an ear tissue biopsy and xenodiagnostic ticks. Challenge nymphs from the single-strain infection treatment were either infected with the focal strain or uninfected. Challenge nymphs from the multiple-strain infection treatment were either infected with strain Fin-Jyv-A3 alone, strain NE4049 alone, infected with both strains, or uninfected.

In a previous study (19, 23), we had infected donor mice via nymphal tick bite (see Section 1 in the supplemental material) with three types of infection: (i) strain Fin-Jyv-A3 alone (*n* = 10 mice), (ii) strain NE4049 alone (*n* = 10 mice), and (iii) infected with both strains (*n* = 20 mice). At 5-week post-infection, we infested the donor mice with naive *I. ricinus* larvae from our laboratory colony (larvae in the top row in Fig. 1). The engorged larvae molted into three types of infected nymphs: (i) putatively infected with strain Fin-Jyv-A3, (ii) putatively infected with strain NE4049, and (iii) putatively infected with both strains (challenge nymphs in the top row in Fig. 1). Not all larvae that feed on an infected mouse acquire the infection and such engorged larvae molt into uninfected nymphs (hence the word ‘putatively’). Larvae that feed on a donor mouse infected with both strains can molt into nymphs with four different infection states: (i) infected with both strains, (ii) infected with strain Fin-Jyv-A3 alone, (iii) infected with strain NE4049 alone, and (iv) uninfected. Unfed nymphs must be alive to infect a mouse, but they must be killed to determine their infection status. This means that we cannot know the infection status of an unfed nymph prior to using it in an infectious challenge. However, we estimated the probability of the three types of infection in our unfed challenge nymphs, by testing a random sample of these unfed nymphs (range = 7–26 per mouse) at 1 month and at 4 months after the larva-to-nymph molt for each of the 40 donor mice (19, 23).

A subsample of 30 donor mice was selected because their unfed nymphs had a high probability of carrying one of the three types of infection of interest (see Section 1 in the supplemental material). In what follows, groups 1, 2, and 3 refer to the unfed nymphs that took their larval blood meal from donor mice infected with strain Fin-Jyv-A3 alone, donor mice infected with strain NE4049 alone, and donor mice infected with both strains, respectively. The nymphal infection prevalence between groups 1, 2, and 3 was 88.2%, 77.0%, and 87.0%, respectively, but the strain composition was of course very different. For the unfed nymphs in group 1, 88.2% (range = 63.6%–100.0% among 9 mice) were infected with strain Fin-Jyv-A3. For the unfed nymphs in group 2, 77.0% (range = 53.3%–100.0% among 9 mice) were infected with strain NE4049. For the unfed nymphs in group 3, 42.4% (range = 12.0%–80.0% among 6 + 6 = 12 mice) were infected with both strains, 14.3% (range = 0.0%–33.0%) were infected with strain Fin-Jyv-A3 alone, 30.3% (range = 8.0%–72.0%) were infected with strain NE4049 alone, and 13.0% were uninfected.

### Nymph-to-host transmission of *B. afzelii*

Hundred female, pathogen-free *M. musculus* BALB/c mice aged 5 weeks were used to estimate the probability of nymph-to-host transmission (bottom row in Fig. 1). The study consisted of two separate, independent experiments where the focal strains were Fin-Jyv-A3 and NE4049, respectively. In experiment 1, test mice were exposed to group 1 nymphs (*n* = 12 mice) or to group 3 nymphs (*n* = 38 mice). In experiment 2, test mice were exposed to group 2 nymphs (*n* = 12 mice) or to group 3 nymphs (*n* = 38 mice). The larger sample size for the group 3 nymphs (2 × 38 = 76 test mice) compared to the group 1 and group 2 nymphs (2 × 12 = 24 test mice) was necessary because the percentage of nymphs infected with both strains in group 3 (42.4%) was lower compared to the percentage of nymphs infected with strain Fin-Jyv-A3 alone (88.2%) and strain NE4049 alone (77.0%). Furthermore, some of the group 3 nymphs were infected with strain Fin-Jyv-A3 alone (14.3%) or strain NE4049 alone (30.3%), and these nymphs would therefore contribute to estimating the probability of nymph-to-host transmission for nymphs infected with single strains.

All 100 test mice were infested with a single challenge nymph aged 10–11 months that was randomly selected from one of the 30 donor mice (bottom row in Fig. 1). Each challenge nymph was placed in a plastic capsule that was attached to the shaved back of the test mouse; the nymphs were checked daily for their attachment success. The challenge nymphs were allowed to feed to repletion on the test mice. The engorged challenge nymphs were collected from the capsules, frozen at −20°C, and tested to determine their strain-specific infection status (bottom row in Fig. 1).

### Ear tissue biopsies, IgG antibodies, and xenodiagnosis to determine *B. afzelii* infection status in the test mice

To confirm *B. afzelii* infection in the test mice, blood and ear tissue samples were taken at 4 weeks after the nymphal challenge. We used quantitative PCR (qPCR) to detect *B. afzelii* in the ear tissue samples of the test mice and SERION ELISA classic *Borrelia burgdorferi* IgG/IgM immunoassay to detect *Borrelia*-specific IgG antibodies in the blood samples of the test mice.

For vector-borne pathogens like *B. burgdorferi* sl, xenodiagnosis is a sensitive method for determining host infection status and involves feeding naive vectors on a host and subsequently testing the vectors for the pathogen (33). We have previously shown that testing of mouse tissues and xenodiagnostic ticks gives identical results with respect to the strain-specific infection status of the mice (10). Five weeks after the nymphal challenge, each test mouse was infested with ~100 pathogen-free xenodiagnostic larval ticks from our laboratory colony of *I. ricinus* (bottom row in Fig. 1). Blood-engorged larvae were kept in individual Eppendorf tubes and were allowed to molt into xenodiagnostic nymphs (bottom row in Fig. 1) under standard laboratory conditions (20°C–25°C, 12 hours light : 12 hours dark). To maintain high humidity, each tube contained a piece of moistened paper towel. Four weeks after the larva-to-nymph molt, 10 xenodiagnostic nymphs were randomly selected for each test mouse and frozen at –20°C for DNA extraction.

### DNA extraction of ticks and mouse tissue samples

The engorged challenge nymphs and the 4-week-old xenodiagnostic unfed nymphs were crushed using a previously described protocol (34). The crushed nymphs were digested with proteinase K at 56°C overnight. The DNA of the nymphs was extracted using QIAGEN DNeasy 96 Blood and Tissue kit well plates and following the QIAGEN protocol. Each plate contained four *B. afzelii*-negative DNA extraction controls (*Anopheles gambiae* mosquitoes) and one water control. DNA from the ear samples of the test mice was extracted using QIAGEN DNeasy Blood & Tissue mini spin columns and following the QIAGEN protocol. Mouse ear tissue DNA and nymph DNA were eluted into 65 µL of water.

### qPCR to determine *B. afzelii* infection status and strain infection status

To determine the *B. afzelii* infection status and the strain infection status of the samples, three different qPCR assays were performed. A qPCR assay that targets a 132-base pair (bp) fragment of the *flagellin* gene was used to determine the *B. afzelii* infection status of the samples (= *flagellin* qPCR). Two strain-specific qPCRs that amplify the same 143-bp fragment of the *ospC* gene, but that use different strain-specific probes that bind to either *ospC* type A3 or A10, were used to identify the strains in the samples (= *ospC* qPCR). Finally, a nested strain-specific qPCR was used to identify the strains in samples that had low abundance of spirochetes (= nested qPCR). First, a PCR that targets a 657-bp fragment of the *ospC* gene (35) was used to amplify the number of *ospC* gene copies. The amplicons were then used as templates in the two strain-specific *ospC* qPCRs to identify the strain infection status of the samples. Unfed nymphs were tested using the *flagellin* qPCR and the two strain-specific qPCRs that target the *ospC* gene. Ear tissue samples from the test mice were tested using the *flagellin* qPCR and the nested qPCR for strain identity. The engorged nymphs were tested with the *flagellin* qPCR, the *ospC* qPCR, and in triplicate with the nested qPCR.

The qPCR assays were done using a LightCycler 96 Multiwell Plate white (Roche). All the plates contained four *B. afzelii*-negative controls for the DNA extraction (mosquito DNA), three negative controls for the qPCR (water), and 12 positive controls at four different dilutions. For each qPCR assay, the 12 positive controls were used to determine the repeatability of the assay. For the *flagellin* qPCR, *ospC* A3 qPCR, and *ospC* A10 qPCR, the repeatability of the log10-transformed spirochete loads was 96.5%, 98.4%, and 96.2%, respectively. The conventional *ospC* PCR (first step of the nested qPCR) was done using a Mastercycler nexus (Eppendorf). All the plates for the conventional PCR contained three negative controls for the PCR (water).

## RESULTS

### *B. afzelii* infection status of the test mice

The *B. afzelii* infection status of the test mice (yes, no) was based on three independent infection phenotypes: presence of *B. afzelii* in the ear punch biopsy, presence of IgG antibodies against *B. afzelii*, and presence of *B. afzelii* in the xenodiagnostic nymphs. We are highly confident in the *B. afzelii* infection status of the test mice because the correspondence between the ear punch biopsies and the xenodiagnostic nymphs was 100.0% (100/100), and their correspondence with the IgG antibody response was 99.0% (99/100). After the nymphal challenge, 60.0% (60/100) of the test mice became infected with *B. afzelii*.

### Strain-specific infection status of the test mice

The strain-specific infection status of the test mice (Fin-Jyv-A3 alone, NE4049 alone, both strains, uninfected) was based on the combined results of the ear punch biopsy and the xenodiagnostic nymphs. The correspondence in the strain-specific infection status between the ear punch biopsy and the xenodiagnostic nymphs was 91.0% (91/100). The remaining nine test mice were all classified as infected with both strains after combining the results for the ear punch biopsy and the xenodiagnostic nymphs.

### Strain-specific infection status of the engorged challenge nymphs

We were able to collect 81 engorged challenge nymphs; the 19 other engorged challenge nymphs could not be found. We compared the strain-specific infection status between the engorged challenge nymphs and the test mice (see Section 2 in the supplemental material for details). For 49 of 81 pairs, there was a perfect match in the strain-specific infection status between the engorged challenge nymph and the test mouse. There were 17 engorged challenge nymphs that tested positive, but the test mice tested negative (i.e., nymphs failed to transmit infection). There were 9 engorged challenge nymphs that tested negative, but the test mice tested positive. There were 6 engorged nymphs that tested positive for a strain that was different from the strain detected in the test mice (these nymphs were all from group 3).

### Effects of the donor mouse infection status and strain on the infection status of the unfed challenge nymphs

**Fig 2:**
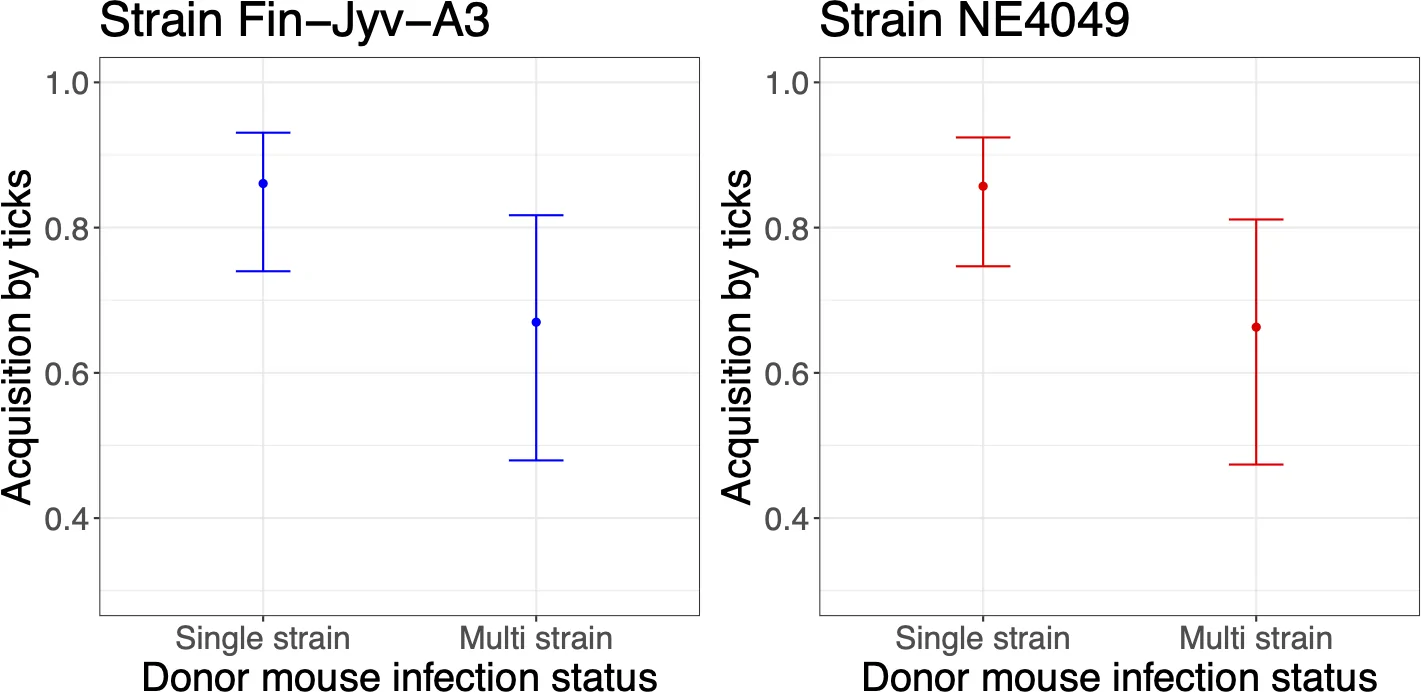
Multiple-strain infection in the donor mice reduced the probability of strain acquisition by unfed *I. ricinus* nymphs for both strains of *B. afzelii*. The proportion of infected unfed challenge nymphs is shown as a function of donor mouse infection status (single strain versus multiple strain) and strain (Fin-Jyv-A3 versus NE4049). The sample sizes for the four groups (from left to right) are 9, 6, 9, and 6 donor mice and 147, 132, 194, and 143 unfed challenge nymphs, respectively (total of 30 donor mice and 616 nymphs). Shown are the means and the 95% confidence intervals.

We had previously shown that multiple-strain infection in the 40 donor mice reduced strain acquisition in the unfed challenge nymphs (note: those studies used the term “host-to-tick transmission” instead of “acquisition”) (10, 19, 23). Here, we wanted to test whether the effects of multiple-strain infection held for the subset of 30 donor mice from which the challenge nymphs were selected. A generalized linear mixed-effects model (GLMM) with binomial errors was used to model the infection status of the unfed challenge nymphs (see Section 3 in the supplemental material for details). The fixed factors included donor mouse infection status (single strain, multiple strain), strain (Fin-Jyv-A3, NE4049), and their interaction; the identity of the donor mice was modeled as a random factor. The interaction between donor mouse infection status and strain was not significant (*P* = 0.055) and was removed from the model. Donor mouse infection status was significant (*P* = 0.013; Fig. 2) but strain was not (*P* = 0.945). Unfed challenge nymphs that had fed as larvae on donor mice infected with both strains had a lower strain acquisition (66.6%) compared to unfed challenge nymphs that had fed as larvae on donor mice infected with a single strain (85.9%; Fig. 2). Thus, multiple-strain infection in the 30 donor mice reduced strain acquisition in the unfed nymphs by 22.4%. If reduced acquisition results in reduced nymph-to-host transmission, we expect that a test mouse infested with one unfed challenge nymph from the multiple strain treatment will be less likely to become infected with the focal strain compared to a test mouse infested with one unfed challenge nymph from the single strain treatment.

### Effects of strain and nymphal age on the strain abundance of the unfed challenge nymphs

We previously showed that multiple-strain infection in the 40 donor mice reduced the spirochete abundance of the strains acquired by the unfed challenge nymphs (19, 23). Here, we restrict the analysis to the subset of nymphs that took their larval blood meal from the donor mice with single-strain infections (i.e., nymphs that took their larval blood meal from multiple-strain infected donor mice were excluded). The spirochete abundance of each strain in the unfed challenge nymphs was estimated by the number of *flagellin* gene copies. A linear mixed-effects model (LMM) was used to analyze the log10-transformed spirochete abundance as a function of strain (Fin-Jyv-A3, NE4049), nymphal age (1 month old, 4 months old), and their interaction; the identity of the donor mice was modeled as a random factor (see Section 4 in the supplemental material for details).

**Fig 3:**
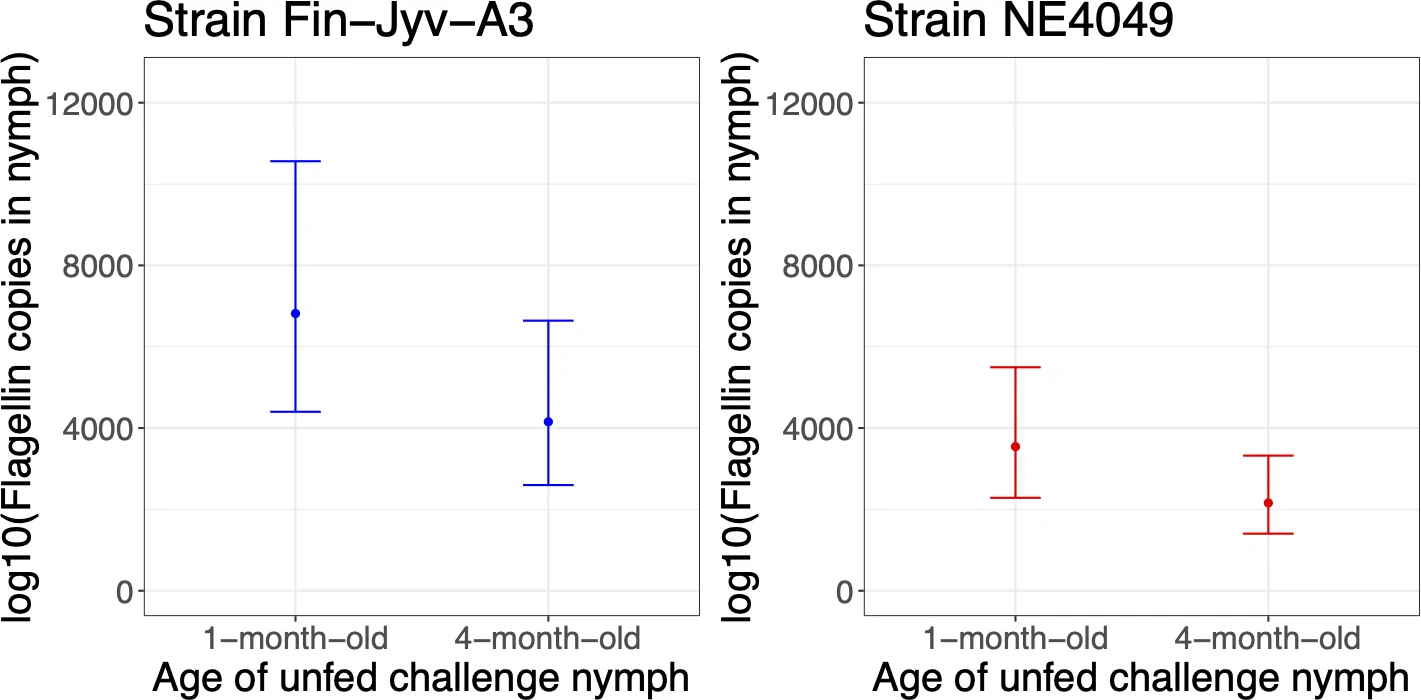
The abundance of strain Fin-Jyv-A3 in the unfed challenge nymphs was 1.9 times higher compared to strain NE4049. The strain abundance in the subset of infected unfed challenge nymphs is shown as a function strain (Fin-Jyv-A3 versus NE4049) and nymphal age (1 month old versus 4 months old). These nymphs fed as larvae on the infected donor mice. For each panel, the sample sizes are based on 9 donor mice that produced both the 1-month-old and the 4-month-old nymphs (a total of 18 donor mice). The four groups (from left to right) are based on 67, 60, 67, and 85 infected unfed challenge nymphs (a total of 279 nymphs). Shown are the means and the 95% confidence intervals.

The interaction between strain and nymphal age was not significant (*P* = 0.608) and was removed from the model. Strain (*P* = 0.016) and nymphal age (*P* = 0.007) both had significant effects on the nymphal strain abundance (Fig. 3). The mean nymphal strain abundance (units = spirochetes per nymph) for strain Fin-Jyv-A3 (mean = 5,321.4) was 1.9 times higher compared to strain NE4049 (mean = 2,766.2). The mean nymphal strain abundance for 1-month-old nymphs (mean = 4,914.9) was 1.6 times higher compared to 4-month-old nymphs (mean = 2,994.9). If strain abundance in the nymph influences nymph-to-host transmission, this result suggests that nymphs infected with strain Fin-Jyv-A3 should have higher nymph-to-host transmission compared to nymphs infected with strain NE4049.

### GLMM to simultaneously test the effects of strain and donor mouse infection status on test mouse infection status

For convenience, we will refer to challenge nymphs that fed as larvae on the donor mice infected with single strains versus both strains as coming from the single strain treatment versus the multiple strain treatment, respectively. Based on our previous analysis of strain acquisition (Fig. 2), we expected that test mice exposed to the nymphs from the single strain treatment will have a higher infection prevalence with that strain compared to test mice exposed to the nymphs from the multiple strain treatment.

**Fig 4:**
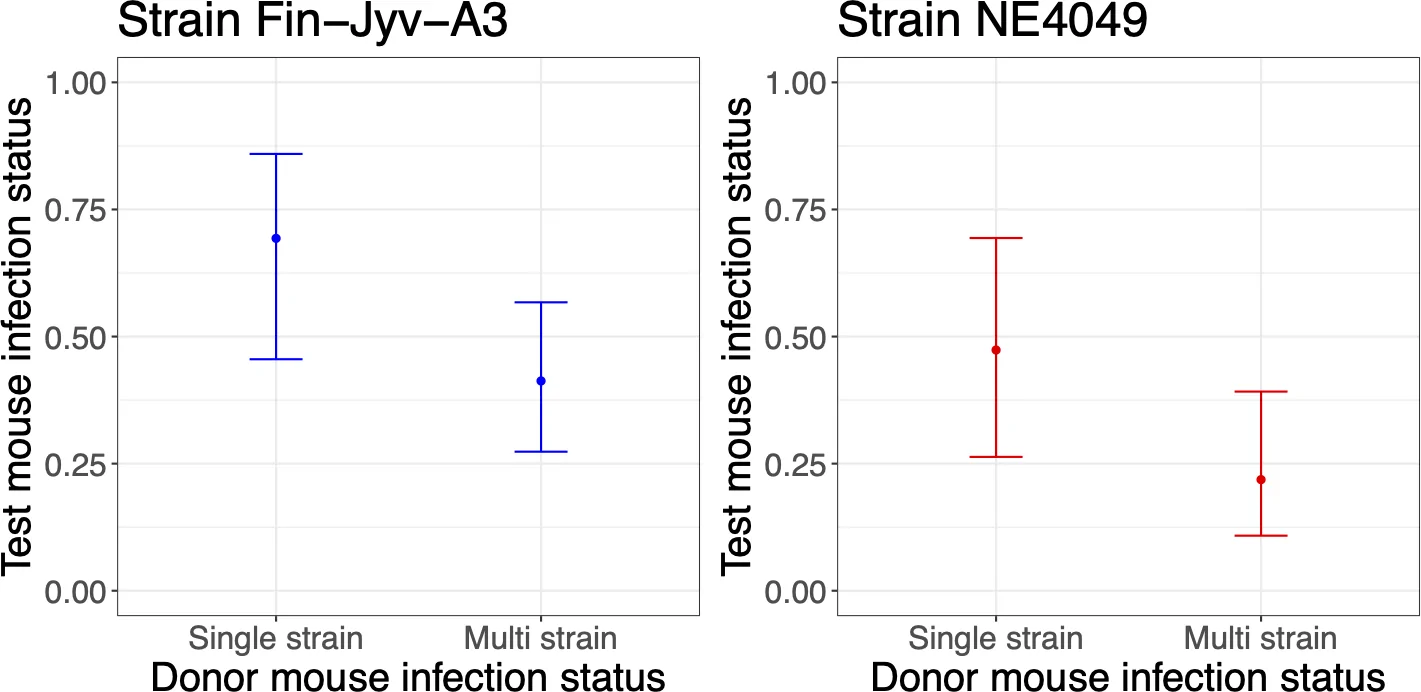
Test mice fed on by putatively infected nymphs from the “multiple strain” group are less likely to be infected with the strain of interest compared to test mice fed on by putatively infected nymphs from the “single strain” group. The proportion of infected test mice is shown as a function of the infection status of the donor mouse (single strain versus multiple strain) that generated the challenge nymph and strain (Fin-Jyv-A3 versus NE4049). Nymphs in the “multiple strain” group took their larval blood meal from donor mice that were infected with both strains (some of these nymphs were not infected or were infected with one of the two strains). Nymphs in the “single strain” group took their larval blood meal from donor mice that were infected with a single strain (some of these nymphs were not infected). The sample sizes for the four groups (from left to right) are 12, 38, 12, and 38 test mice (a total of 100 test mice). Shown are the means and the 95% confidence intervals.

A GLMM with binomial errors was used to analyze the infection status of the 100 test mice as a function of donor mouse infection status (i.e., that produced the challenge nymphs), strain, and their interaction; the identity of the donor mice was modeled as a random factor (see Sections 5 and 6 in the supplemental material for details). The interaction between donor mouse infection status and focal strain was not significant (*P* = 0.753) and was therefore removed from the model. The infection status of the donor mouse (*P* = 0.031) and strain (*P* = 0.049) were both significant (Fig. 4; Table 1).

**TABLE 1:** Parameter estimates^*a*^

Part	Parameter	Fixed effects	Estimate	SE	*z*	*P*
A	Intercept	Reference^*b*^	0.815	0.507	1.607	0.108
A	Contrast	Multiple–single	−1.168	0.540	−2.164	0.031
A	Contrast	NE4049–Fin-Jyv-A3	−0.921	0.467	−1.971	0.049

Part	Donor mouse	Strain	Mean (%)	LL (%)	UL (%)
B	Single strain		58.8	38.0	76.8
B	Multiple		30.7	19.9	44.1
B		Fin-Jyv-A3	55.7	39.9	70.5
B		NE4049	33.4	19.9	50.3
B	Single	Fin-Jyv-A3	69.3	45.5	85.9
B	Multiple	Fin-Jyv-A3	41.3	27.3	56.7
B	Single	NE4049	47.4	26.3	69.4
B	Multiple	NE4049	21.9	10.8	39.2

^*a*^ (A) Parameter estimates on the logit scale are shown for the GLMM of the infection status of the test mice. The model included the fixed factors of donor mouse infection status (single strain, multiple-strain infection) and *B. afzelii* strain (Fin-Jyv-A3, NE4049). For each parameter, the estimate, standard error (SE), *z*-value (*z*), and *P*-value (*P*) are shown. (B) The parameter estimates were back-transformed to the original probability scale (%). For each combination of donor mouse infection status and strain, the mean proportion of infected test mice, lower limit (LL), and upper limit (UL) of the 95% confidence interval are shown.

^*b*^ Reference = Test mouse was infested with a challenge nymph that obtained its larval blood meal from a donor mouse infected with a single strain, which was Fin-Jyv-A3.

Nymphs from the single strain treatment (i.e., includes uninfected nymphs) infected 58.8% of the test mice with the focal strain, whereas nymphs from the multiple strain treatment (i.e., includes uninfected nymphs and nymphs infected with the competitor strain) infected 30.7% of the test mice with the focal strain (Fig. 4; Table 1). Thus, nymphs from the single strain treatment were 1.9 times more likely to infect the test mice with the focal strain compared to nymphs from the multiple strain treatment. This result is expected because for the single strain group, all infected nymphs carry the focal strain, whereas for the multiple strain group, a substantial fraction of infected nymphs carries the competitor strain and not the focal strain. Nevertheless, this result is important because it shows that the 22.4% reduction in strain acquisition for the nymphs in the multiple strain group (Fig. 2) subsequently reduced the strain infection success in the test mice by 47.8% (Fig. 4). In other words, the observed reduction in strain acquisition in the nymphs (measured by qPCR in 1-month-old and 4-month-old nymphs) had real and negative consequences for strain fitness when those same nymphs (at 10 months of age) were fed on the test mice. Nymphs putatively infected with strain Fin-Jyv-A3 versus strain NE4049 (i.e., includes uninfected nymphs) infected 55.7% versus 33.4% of the test mice, respectively (Fig. 4; Table 1).

As pointed out by Eisen (36), estimates of the strain-specific probability of nymph-to-host transmission should be calculated for the subset of hosts that were challenged with a nymph carrying the strain of interest; hosts challenged with an uninfected nymph (or with nymphs carrying the wrong strain) should be excluded (36). The previous analysis did not estimate the effects of multiple-strain infection in the nymph on the probability of strain-specific nymph-to-host transmission because they contained uninfected nymphs as well as nymphs that were singly infected with the focal strain after feeding on donor mice infected with both strains.

### Effect of strain and infection status of the engorged challenge nymph on test mouse infection status

For 81 test mice, the challenge nymph was recovered, and its infection status was determined. For this sample of 81 test mice, we can test whether multiple-strain infection in the nymph and strain identity influence the probability of nymph-to-host transmission. AIC-based model selection and model simplification converged on the same best model (see Sections 7 and 8 in the supplemental material for details). A GLMM with binomial errors was used to analyze the test mouse infection status as a function of strain, and the infection status of the engorged nymph with respect to the focal strain and the competitor strain. The presence of the competitor strain in the engorged nymph had no effect on the nymph-to-host transmission of the focal strain (*P* = 0.099). Strain identity (*P* = 0.002) and whether the engorged nymph tested positive or negative for the focal strain (*P* < 0.001) were both significant with respect to nymph-to-host transmission (Table 2).

**TABLE 2:** Parameter estimates^*a*^

Part	Parameter	Fixed effects	Estimate	SE	*z*	*P*
A	Intercept	Reference^*b*^	−1.076	0.310	−3.467	<0.001
A	Contrast	NE4049–Fin-Jyv-A3	−1.223	0.404	−3.030	0.002
A	Slope	Infection status	2.421	0.402	6.021	<0.001

Part	Strain	Engorged nymph	Mean (%)	LL (%)	UL (%)
B	Fin-Jyv-A3	Uninfected	25.4	15.7	38.5
B	Fin-Jyv-A3	Infected	79.3	65.7	88.5
B	NE4049	Uninfected	9.1	4.5	17.8
B	NE4049	Infected	53.0	37.5	68.0

^*a*^ (A) Parameter estimates on the logit scale are shown for the GLMM of the infection status of the test mice. The model included the fixed factors of strain (Fin-Jyv-A3, NE4049) and infection status of the engorged nymph with respect to the focal strain (uninfected, infected). For each parameter, the estimate, SE, z-value (*z*), and *P*-value (*P*) are shown. (B) The parameter estimates were back-transformed to the original probability scale (%). For each combination of strain and infection status of the engorged nymph with respect to the focal strain, the mean probability of nymph-to-host transmission, SE, lower limit (LL), and upper limit (UL) of the 95% confidence interval are shown.

^*b*^ Reference = Fin-Jyv-A3 is the focal strain, and the engorged nymph is uninfected with the focal strain.

**Fig 5:**
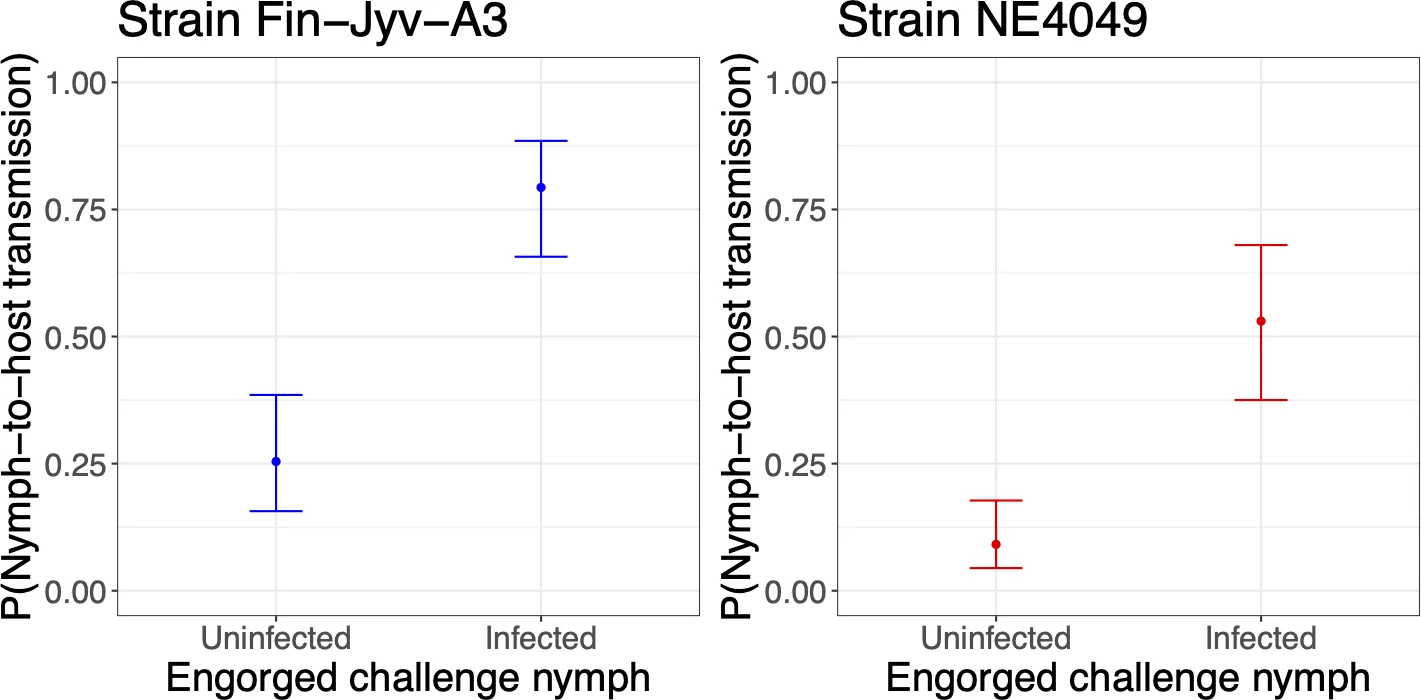
Probability that the test mouse is infected depends on the strain (Fin-Jyv-A3 and NE4049) and the infection status of the engorged challenge nymph (uninfected, infected) with respect to the focal strain. The analysis is restricted to the subset of 81 test mice for which the engorged challenge nymph was recovered. As a reminder, because not all larvae fed on an infected host become infected, and because there is no way to check the infection status of a challenge nymph before it feeds on a host, the infection status of a challenge nymph, when checked as an engorged nymph, may end up being uninfected. For the infected challenge nymphs, the proportion of infected test mice estimates the probability of nymph-to-host transmission for the two strains. The sample sizes for the four combinations (from left to right) are 45, 36, 50, and 31 test mice (total of 2 × 81 = 162 test mice; each of the 81 test mice appears twice in the analysis, once for each strain). Shown are the means and the 95% confidence intervals.

As expected, engorged challenge nymphs that tested positive for the focal strain infected a higher percentage of test mice compared to engorged challenge nymphs that tested negative for the focal strain (Fig. 5; Table 2). Averaged across the two strains, nymph-to-host transmission for infected engorged nymphs (67.6%) was 4.3 times higher compared to uninfected engorged nymphs (15.6%). Nymph-to-host transmission was higher for strain Fin-Jyv-A3 compared to strain NE4049 (Fig. 5; Table 2). If the engorged challenge nymph was infected with the focal strain, nymph-to-host transmission of strain Fin-Jyv-A3 (79.3%) was 1.5 times higher compared to strain NE4049 (53.0%). Thus, nymphs infected with strain Fin-Jyv-A3 are considerably more infectious for BALB/c mice compared to nymphs infected with strain NE4049.

## DISCUSSION

### Effects of multiple-strain infection in the rodent host on the fitness of *B. afzelii* strains

We and others have previously shown that infection with multiple strains of *B. burgdorferi* sl in the rodent host reduced strain acquisition in ticks that fed on those hosts (note: those studies used the term “host-to-tick transmission” instead of “acquisition”) (19, 20, 23, 37). The negative effect of multiple-strain infections in the host on strain acquisition in ticks has been observed in both laboratory mice (*Mus musculus*) (19, 23) and natural reservoir hosts such as the white-footed mouse (*Peromyscus leucopus*) (20, 37). In the present study, we confirmed that the probability that the unfed nymphs from the multi-strain treatment (group 3) carried the focal strain was reduced by 23.2% compared to unfed nymphs from the single strain treatments (groups 1 and 2) (Fig. 2). Here we show for the first time that this reduction in strain acquisition resulted in a 45.8% reduction in the percentage of hosts infected with the focal strain in the next step of the life cycle (Fig. 4). Challenge nymphs from the multiple strain treatment infected a lower percentage of test mice with the focal strain compared to challenge nymphs from the single strain treatment (30.7% versus 58.8%; Table 1; Fig. 4). The explanation is simple—the strain composition was different between nymphs from the single strain group and the multiple strain group (even though the *B. afzelii* infection prevalence was the same). For the single strain group, all infected nymphs carried the focal strain of interest, whereas for the multiple strain group, a substantial fraction of infected nymphs carried the “wrong” competitor strain rather than the “right” focal strain (Fig. 2). Although expected, this result shows that our qPCR-based estimates of strain acquisition in nymphs do predict their future strain-specific probability of nymph-to-host transmission. In summary, our study shows that multiple-strain infection in the donor mice (and the associated reduction in strain acquisition by ticks) has a significant negative effect on the fitness of these strains in the next step of their life cycle (i.e., because nymphs carrying the wrong competitor strain cannot infect the test mice with the right focal strain).

### Effects of multiple-strain infection in the nymphal tick on the fitness of *B. afzelii* strains

We tested whether multiple-strain infection in the nymph influenced nymph-to-host transmission of the strains by including the infection status of the engorged nymph with respect to the focal strain and the competitor strain as covariates. As expected, the test mouse was more likely to become infected with the focal strain if the engorged challenge nymph tested positive for the focal strain. The presence of a competitor strain in the engorged challenge nymph had no effect on the probability of nymph-to-host transmission of the focal strain. This result suggests that multiple-strain infection in the nymph does not result in competition with respect to strain-specific nymph-to-host transmission. This finding was unexpected because we had previously shown that multiple-strain infection with strains Fin-Jyv-A3 and NE4049 in *I. ricinus* nymphs reduces the spirochete load of each strain by ~50% (19, 23). If the nymphal spirochete load is important for nymph-to-host transmission, we expect that multiple-strain infected nymphs would have lower nymph-to-host transmission for each strain compared to nymphs infected with single strains. One explanation is that even though multiple-strain infection in the nymphs reduced the strain-specific spirochete load by 50% (19, 23), the absolute numbers of spirochetes were still sufficiently high to maintain effective nymph-to-host transmission for both strains.

Our finding that multiple-strain infection in the nymph had no effect on nymph-to-host transmission of each strain is important because in nature, wild *Ixodes* nymphs are often infected with multiple strains of *B. burgdorferi* sl pathogens (30, 31, 38). Numerous studies have suggested that the nymph represents a bottleneck for the strain diversity of *B. burgdorferi* sl and other tick-borne pathogens (20, 21, 39, 40). However, to date, there are no data on how multiple-strain infection in *Ixodes* nymphs influences strain-specific nymph-to-host transmission. A theoretical model that investigated the co-existence of two *B. burgdorferi* sensu stricto (ss) strains in nature assumed that nymphs carrying two strains transmitted only one of the two strains to the rodent reservoir host during the nymphal blood meal (20). Another theoretical model assumed that if more strains are present in the nymph, each strain is less likely to be transmitted to the host (40). The present study demonstrates that for nymphs carrying two *B. afzelii* strains, each strain acts independently to infect the rodent reservoir host during the nymph-to-host transmission step. Future studies should investigate whether nymph-to-host transmission is reduced when nymphs are infected with three or more strains.

### Estimates of nymph-to-host transmission from experimental infection studies

In experimental infection studies, the probability that the host will become infected with the tick-borne pathogen following the tick challenge is determined by the equation *P*(infection) = 1 – (1 – *P*)^*n*^, where *n* is the number of ticks used to challenge the host and *P* is the probability that those ticks are infected with the pathogen of interest. For example, if 50% of the challenge nymphs are infected (*P* = 0.50), then *P*(infection) is 50.0% for a challenge with one nymph and 87.5% for a challenge with three nymphs. A study on *Borrelia mayonii* in *Ixodes scapularis* found that the probability that the host became infected was 40% for infestations with single nymphs, whereas it was 83.3% for infestations with two nymphs (41). In the present study on *B. afzelii* strain NE4049, the probability that a test mouse became infected following infestation with a single nymph was 53.0% (Table 2), whereas a previous experimental infection study with this strain, where each rodent host was infested with three challenge nymphs, found that the probability of infection was >95% (42). A qualitative review of the literature suggests that the probability of infection for the host following a tick challenge is generally higher for infestations with multiple nymphs per host compared to infestations with single nymphs per host (see Section 9 in the supplemental material for details). Most infection studies challenge hosts with multiple nymphs because the purpose is to create infected hosts, but this approach makes it difficult to determine the probability of nymph-to-host transmission per infected tick bite (42 – 44). In summary, infestations with single nymphs are the most efficient experimental design for estimating the strain-specific probability of nymph-to-host transmission.

Another complicating factor for infecting hosts via tick bite is that the percentage of ticks that are infected with the pathogen of interest can vary dramatically. For example, in a study that compared nymph-to-host transmission success among strains of *B. afzelii*, the probability that the challenge nymph was infected with the strain of interest ranged from 15.4% to 100.0% (44). The probability of nymph-to-host transmission must be calculated for the subset of hosts exposed to a single infected challenge nymph, and it is therefore critical to retrospectively test the infection status of the engorged nymphs after the infectious challenge. In the present study, estimates of nymph-to-host transmission for strains Fin-Jyv-A3 and NE4049 for the subset of mice exposed to an infected challenge nymph (79.3% and 53.0%; Table 2; Fig. 5) were considerably higher compared to the estimates that also included uninfected challenge nymphs (55.7% and 33.4%; Table 1). This example demonstrates the importance of determining the infection status of the challenge nymph to obtain the best estimates of the probability of nymph-to-host transmission.

### *B. afzelii* strains differ in their probability of nymph-to-host transmission

The probability of nymph-to-host transmission is the probability that the host becomes infected with the pathogen after having been exposed to a single infected challenge nymph that fed to repletion. In the present study, we found that the probability of nymph-to-host transmission for strain Fin-Jyv-A3 (79.3%; Table 2) was 1.5 times higher compared to strain NE4049 (53.0%; Table 2; Fig. 5). To our knowledge, this is the first study to demonstrate that strains can differ in their nymph-to-host transmission. Other studies on *B. afzelii* and *B. burgdorferi* ss have compared nymph-to-host transmission among strains, but the sample sizes were too small to draw valid conclusions (45, 46). Our study demonstrates that large sample sizes of test mice (*n* = 50 per strain) are required to detect significant differences in nymph-to-host transmission between strains. All else being equal, the observed 1.5-fold difference in nymph-to-host transmission would result in major differences in fitness between the two strains; strain Fin-Jyv-A3 would be expected to have a higher frequency in nature compared to strain NE4049.

A limitation of this study is that we have no understanding of the genes that are responsible for differences in nymph-to-host transmission between these two strains of *B. afzelii*. We know that these two strains differ with respect to their MLST and *ospC* type. We had previously shown that these two strains are very similar with respect to their plasmid profile (10), which resembles that of other *B. afzelii* strains (47). Both strains share 11 linear plasmids (lp17, lp28-2, lp28-3, lp25, lp28, lp28-4, lp28-7, lp28-8, lp30, lp34, and lp38) and three circular plasmids (cp26, cp32-9, and cp32-10). Strain Fin-Jyv-A3 but not strain NE4049 has cp32-5, and both strains are missing cp32-7 (10). These plasmids carry many critical virulence genes that are essential for *B. burgdorferi* sl pathogens to establish infection in the vertebrate host and the tick vector (48 – 52). For example, some plasmids carry genes that code for outer surface proteins that allow *B. burgdorferi* sl pathogens to evade the complement system of the vertebrate host (53 – 55). Other plasmid-encoded adhesins allow the spirochete to bind to molecules in the tick midgut (51, 56, 57) or to the extracellular matrix components of the connective tissues of the vertebrate host (48, 49, 58). Nymph-to-host transmission requires colonization and persistence of spirochetes in the midgut of the nymphal tick, migration to tick salivary glands, inoculation into the host, evasion of host complement, followed by dissemination and colonization of distant host tissues. In summary, nymph-to-host transmission is influenced by many different plasmid-encoded virulence factors.

### Nymphal spirochete load and nymph-to-host transmission of *B. burgdorferi* sl

The mechanism underlying the higher nymph-to-host transmission of strain Fin-Jyv-A3 compared to strain NE4049 may be related to bacterial abundance in the nymph. This idea is supported by a field study on *I. ricinus*, which found that *B. afzelii* strains that established a high spirochete load in nymphs had higher frequency in the pathogen population (31). When *Ixodes* nymphs begin their blood meal, spirochetes replicate in the midgut of the nymphs and migrate to the salivary glands for transmission to the host (51, 59 – 61). In the present study (Fig. 3) and previous studies, we have shown that strain Fin-Jyv-A3 has a nymphal spirochete load that is approximately two times higher compared to strain NE4049 (19, 23). Thus, the higher nymphal spirochete loads of strain Fin-Jyv-A3 may explain its higher nymph-to-host transmission compared to strain NE4049. An alternative explanation is that strain Fin-Jyv-A3 is better than strain NE4049 at persisting in the nymphal midgut, evading the tick immune system, and invading the tick salivary glands (51, 62, 63). Future studies should investigate whether differences in nymphal spirochete load between strains explain their differences in nymph-to-host transmission.

### Age of the nymph and nymph-to-host transmission of *B. burgdorferi* sl

The age of the nymph may also influence the probability of nymph-to-host transmission of *B. burgdorferi* sl pathogens. In the present study (Fig. 3) and previous studies, we have shown that the nymphal spirochete load decreases with nymphal age, and we have speculated that the spirochete viability and the probability of nymph-to-host transmission decrease accordingly (19, 23, 64, 65). In the present study, the nymphs were 10 months old at the time of the nymphal challenge and the nymph-to-host transmission of strain NE4049 was 53.0%. In a previous study on *B. afzelii* strain NE4049 in the same mouse strain (BALB/c), the nymphs were 1 month old at the time of the nymphal challenge and the nymph-to-host transmission was 100.0% (43). Thus, 1-month-old nymphs are more infectious to rodent hosts compared to 10-month-old nymphs, and one explanation is that the viability of the spirochetes decreases over time as the nymphs age.

### Maintenance of *B. afzelii* strain diversity in nature

In nature, populations of *B. burgdorferi* sl often consist of a dozen strains carrying different *ospC* types that circulate in the same vertebrate host and tick populations at relatively small spatial scales (8, 14, 30, 31, 33, 66 – 69). We expect competition between strains that share a common niche to result in the loss of strain diversity over time (4). However, long-term studies of strain diversity in populations of *B. afzelii* have shown that the strain composition is relatively constant over time (66, 67). This long-term stability raises the obvious question of what ecological factors facilitate the maintenance of strain diversity in *B. burgdorferi* sl populations. The two main explanations for the maintenance of this strain diversity are the multiple niche polymorphism (MNP) hypothesis and the negative frequency–dependent selection (NFDS) hypothesis coupled with antigenic diversity at the *ospC* gene (14, 33, 40, 69 – 71). Under the MNP hypothesis, different strains are adapted to different vertebrate host species (33, 40, 55, 70, 72, 73). Evidence for the MNP hypothesis for *B. burgdorferi* sl consists of field studies that have searched for statistical associations between strains and vertebrate host species (33, 66, 72 – 74). For *B. burgdorferi* ss, some studies have found associations (33, 72) and others have not (73, 74). For *B. afzelii*, a 9-year field study that sampled seven *ospC* strains from 2,800 animals belonging to three host species found no evidence for the MNP hypothesis (66). Under the NFDS hypothesis, vertebrate hosts are more likely to encounter and develop antibody responses against common *ospC* types, which subsequently selects against strains carrying those *ospC* types and reduces their transmission and frequency (14, 40, 69, 70, 75, 76). A recent theoretical model showed that MNP and NFDS may both play a role in the maintenance of the strain diversity of *B. burgdorferi* sl pathogens in nature (40).

Our study suggests that strain-specific adaptations to different steps of the pathogen life cycle could also help maintain pathogen strain diversity in nature. Life history theory hypothesizes that vector-borne pathogens should experience trade-offs in fitness or performance between the vertebrate host and the arthropod vector (3, 44). The evidence that strains Fin-Jyv-A3 and NE4049 have superior performance in the tick vector versus the rodent host is as follows. Strain Fin-Jyv-A3 establishes higher spirochete loads in nymphal ticks (19, 23) and has higher nymph-to-host transmission compared to strain NE4049. In contrast, in mice infected with both strains, strain NE4049 infected more mouse tissues, which resulted in higher acquisition in ticks compared to strain Fin-Jyv-A3 (10). Our work suggests that strain NE4049 is better adapted to the rodent host, whereas strain Fin-Jyv-A3 is better adapted to the tick vector.

### Infection status of engorged nymphs

As expected, the strain-specific infection status of the engorged challenge nymphs was a good predictor of the strain-specific infection status of the test mice. In 60.5% of the cases (49/81), there was a perfect match in the strain-specific infection status between the engorged challenge nymph and the test mouse. In 39.5% of the cases (32/81), the strain-specific infection status of the engorged challenge nymph was different from the strain-specific infection status of the test mouse. For example, engorged nymphs that tested positive for *B. afzelii* strains Fin-Jyv-A3 and NE4049 failed to infect 20.7% and 47.0% of the test mice, respectively (Table 2). One explanation is that the spirochete population in some of these 10- to 11-month-old challenge nymphs was no longer viable or was too small to establish infection in the test mice. Conversely, 25.4% and 9.1% of test mice became infected with Fin-Jyv-A3 and NE4049, respectively (Table 2), even though their engorged nymphs tested negative. A recent study on the kinetics of *B. afzelii* in *I. ricinus* found that the number of spirochetes in the nymph decreased by >90% by the third day of the nymphal blood meal (nymphal spirochete loads decreased from 10,907 prior to feeding to 720 by the third day of blood intake) (65). Thus, one explanation for the high percentage of engorged nymphs that tested negative in our study but that still infected the test mice is that the spirochete loads in the engorged nymphs dropped below the level of detection of our qPCR assay during the nymphal blood meal. We point out that the kinetics of *B. afzelii* in *I. ricinus* (65) is different from *B. burgdorferi* ss in *I. scapularis* where the spirochete population increases dramatically during the nymphal blood meal (60, 62, 77). In summary, the infection status of the engorged nymph was generally but not always predictive of the infection status of the test mouse.

### Conclusions

Multiple-strain infection in the rodent host reduced the acquisition of *B. afzelii* strains by immature *I. ricinus* ticks. An important result is our demonstration that this reduction in strain acquisition had real fitness consequences when the resultant challenge nymphs fed on the test mice in the next step of the life cycle. We found no evidence that multiple-strain infection in the nymph influenced the probability of nymph-to-host transmission of either strain. This result suggests that in nymphs carrying both strains, the two strains essentially behave independently of each other during the nymphal blood meal. Strain Fin-Jyv-A3 had a nymphal spirochete load that was 1.9 times higher compared to strain NE4049. Nymph-to-host transmission of strain Fin-Jyv-A3 was 1.5 times higher compared to strain NE4049. These results suggest that nymphal spirochete load might determine the probability of nymph-to-host transmission for *B. afzelii* and perhaps other *B. burgdorferi* sl pathogens.

## Data Availability

The data sets presented in this manuscript were uploaded as Excel files to Zenodo with the following DOI: 10.5281/zenodo.7958506.

## SUPPLEMENTAL MATERIAL

Supplemental statistical analyses spectrum.01675-23-s0001.pdf This supplemental material contains 9 sections that contain the statistical analyses that support the main manuscript.

ASM does not own the copyrights to Supplemental Material that may be linked to, or accessed through, an article. The authors have granted ASM a non-exclusive, world-wide license to publish the Supplemental Material files. Please contact the corresponding author directly for reuse.
