# Supplementary material for: Tick-to-host transmission differs between Borrelia afzelii strains
Source: Microbiol Spectr. 2023 Sep 7;11(5):e01675-23. doi: 10.1128/spectrum.01675-23 (PMC10580945; doi:10.1128/spectrum.01675-23)
Supplement: Supplemental statistical analyses — This supplemental material contains 9 sections that contain the statistical analyses that support the main manuscript. [file spectrum.01675-23-s0001.pdf]

Supplementary material

Title: Tick-to-host transmission differs between *Borrelia afzelii* strains

Dolores Genné, Whitney Jiricka, Anouk Sarr, Maarten J. Voordouw

Table of Contents

Section 1 – Creation of the challenge nymphs ... 2

Section 2 – Correspondence between the strain-specific infection status of the engorged challenge nymphs and the test mice ... 4

Section 3 – Effects of the donor mouse infection status and strain on the infection status of the unfed challenge nymphs ... 7

Section 4 – Effects of strain and nymphal age on the strain abundance of the unfed challenge nymphs ... 8

Section 5 – GLMM to simultaneously test the effects of strain and donor mouse infection status on test mouse infection status ... 11

Section 6 – Proportion tests to separately compare the effects of donor mouse infection status and strain on the infection prevalence of the test mice ... 14

Section 7 – Infection status of the engorged challenge nymph is the best predictor of the infection status of the test mice ... 15

Section 8 – Effect of strain and infection status of the engorged challenge nymph on test mouse infection status ... 17

Section 9 – Literature review of estimates of nymph-to-host transmission of *B. burgdorferi* sl pathogens ... 19

Section 1 – Creation of the challenge nymphs

Borrelia afzelii strains Fin-Jyv-A3 and NE4049 were cultured in BSK-H media at 34 °C. Each of 5 specific pathogen-free female BALB/c mice were inoculated with 1.0 x 10^6 spirochetes in 100 μL of 1x PBS. At four weeks post-infection, these needle-infected mice were infested with ~100 *Ixodes ricinus* larvae from our specific pathogen-free colony at the University of Neuchatel. Engorged larval ticks were placed in individual Eppendorf tubes and were allowed to molt into nymphs under standard laboratory conditions (20–25 °C, 12 h light : 12 h dark). To maintain high humidity, each tube contained a piece of moistened paper towel. At four weeks after the larva-to-nymph molt, a random sample of nymphs was selected for each strain and tested for *B. afzelii* infection using qPCR. The percentage of nymphs infected with *B. afzelii* was 70% (7/10) for strain Fin-Jyv-A3 and 71.4% (10/14) for strain NE4049. These nymphs were used to infect the donor mice in the present study.

The infection of the donor mice via nymphal tick bite has been described previously (1, 2). Briefly, 40 female BALB/c mice, referred to as the donor mice in the present study, were randomly assigned to one of four infection treatments: (1) Fin-Jyv-A3 single strain (n = 10), (2) multiple strain infection with Fin-Jyv-A3 and NE4049 (n = 10), (4) NE4049 single strain (n = 10), and (4) multiple strain infection with NE4049 and Fin-Jyv-A3 (n = 10). Donor mice in the single strain treatments were infested with 5 nymphs putatively infected with the stain of interest. Donor mice in the multiple strain infection treatments were infested with 5 nymphs putatively infected with Fin-Jyv-A3 and 5 nymphs putatively infected with strain NE4049. Five weeks after the nymphal challenge, each donor mouse was infested with ~100 *I. ricinus* larvae from our specific pathogen-free colony at the University of Neuchatel (i.e., the larvae in top row titled 'Previous Study' of Figure 1 in the main manuscript). Blood-engorged larvae were allowed to molt into nymphs as described above (i.e., the nymphs in top row titled 'Previous Study' of Figure 1 in the main manuscript). These nymphs were used as the challenge nymphs in the current study (i.e., the nymphs in bottom row titled 'Current Study' of Figure 1 in the main manuscript).

Table S1. For the second part of the study, a subset of 30 donor mice was selected from the 37 donor mice because their unfed nymphs had a high probability of carrying the three types of infection of interest. For each of the 30 donor mice, we tested a random sample of unfed nymphs to determine the probability that these nymphs carried the three types of infection of interest. For the 30 donor mice, this table shows their unique ID number (Mouse ID), focal strain (Focal strain: Fin-Jyv-A3 or NE4049), infection treatment (Infection: Single, Multiple), number of challenge nymphs that tested positive with strain Fin-Jyv-A3 alone (Fin-Jyv-A3), number of challenge nymphs that tested positive with strain NE4049 alone (NE4090), number of challenge nymphs that were infected with both strains (Multiple), number of challenge nymphs that were uninfected (Uninf), and total number of challenge nymphs that were tested (Total).

Mouse ID	Focal Strain	Infection	Fin-Jyv-A3	NE4049	Multiple	Uninf	Total
S01	Fin-Jyv-A3	Single	8	0	0	0	8
S02	Fin-Jyv-A3	Single	9	0	0	0	9
S03	Fin-Jyv-A3	Single	22	0	0	2	24
S04	Fin-Jyv-A3	Single	20	0	0	2	22
S06	Fin-Jyv-A3	Single	14	0	0	8	22
S07	Fin-Jyv-A3	Single	22	0	0	1	23
S08	Fin-Jyv-A3	Single	19	0	0	5	24
S09	Fin-Jyv-A3	Single	6	0	0	1	7
S10	Fin-Jyv-A3	Single	7	0	0	1	8
S11	Fin-Jyv-A3	Multiple	1	18	3	3	25
S13	Fin-Jyv-A3	Multiple	1	1	8	0	10
S14	Fin-Jyv-A3	Multiple	5	5	9	4	23
S15	Fin-Jyv-A3	Multiple	0	14	5	5	24
S16	Fin-Jyv-A3	Multiple	7	5	12	1	25
S19	Fin-Jyv-A3	Multiple	6	2	13	4	25
S30	NE4049	Single	2	11	4	7	24
S31	NE4049	Single	0	17	0	2	19
S32	NE4049	Single	0	19	0	5	24
S33	NE4049	Single	0	26	0	0	26
S34	NE4049	Single	0	10	0	4	14
S35	NE4049	Single	0	15	0	9	24
S36	NE4049	Single	0	23	0	1	24
S38	NE4049	Single	0	8	0	7	15
S39	NE4049	Single	0	17	0	7	24
S40	NE4049	Single	0	17	0	7	24
S21	NE4049	Multiple	2	11	3	6	22
S22	NE4049	Multiple	0	6	18	1	25
S23	NE4049	Multiple	0	7	15	2	24
S24	NE4049	Multiple	8	4	10	2	24
S28	NE4049	Multiple	8	2	12	2	24

Section 2 – Correspondence between the strain-specific infection status of the engorged challenge nymphs and the test mice

Correspondence of strain-specific infection status of engorged challenge nymphs in group 1 and their test mice: There were 12 test mice that were challenged with group 1 nymphs that were putatively infected with strain Fin-Jyv-A3. Following the infectious challenge, the engorged challenge nymphs and the test mice were tested for their infection status. The correspondence in infection status between the engorged challenge nymphs and the test mice is shown in **Table S2**. We recovered 9 of the 12 engorged challenge nymphs (i.e., 3 engorged challenge nymphs were not recovered). Of the 9 recovered engorged nymphs, the infection status of 7 engorged nymphs was congruent with their test mice (highlighted in green in **Table S2**). There were 2 engorged nymphs that tested positive for strain Fin-Jyv-A3, but these nymphs failed to transmit the infection to the mice (highlighted in blue in **Table S2**).

Table S2. This contingency table shows the correspondence between the infection status of the engorged challenge nymphs (rows) from group 1 and the test mice (columns). Group 1 challenge nymphs took their larval blood meal from donor mice infected with strain Fin-Jyv-A3 alone and therefore have two possible states: infected with strain Fin-Jyv-A3 or uninfected. The row titled 'Unrecovered' refers to engorged nymphs that were not recovered and were therefore not tested with respect to their strain-specific infection status.

	Infection status of mice		
Infection status of engorged nymphs	Fin-Jyv-A3	Uninfected	Total
Fin-Jyv-A3	6	2	8
Uninfected	0	1	1
Unrecovered	2	1	3
Total	8	4	12

Correspondence of strain-specific infection status of engorged challenge nymphs in group 2 and their test mice: There were 12 test mice that were challenged with group 2 nymphs that were putatively infected with strain NE4049. The correspondence in infection status between the engorged challenge nymphs and the test mice is shown in **Table S3**. We recovered 9 of the 12 engorged challenge nymphs (i.e., 3 engorged challenge nymphs were not recovered). Of the 9 recovered engorged nymphs, the infection status of 3 engorged nymphs was congruent with their test mice (highlighted in green in **Table S3**). There was 1 nymph that tested negative for strain NE4049 but that did transmit the infection to the test mouse (highlighted in yellow in **Table S3**). There were 5 engorged nymphs that tested positive for strain NE4049, but these nymphs failed to transmit the infection to the mice (highlighted in blue in **Table S3**).

Table S3. This contingency table shows the correspondence between the infection status of the engorged challenge nymphs (rows) from group 2 and the test mice (columns). Group 2 challenge nymphs took their larval blood meal from donor mice infected with strain NE4049 alone and therefore have two possible states: infected with strain NE4049 or uninfected. The row titled 'Unrecovered' refers to engorged nymphs that were not recovered and were therefore not tested with respect to their strain-specific infection status.

	Infection status of mice		
Infection status of engorged nymphs	NE4049	Uninfected	Total
NE4049	3	5	8
Uninfected	1	0	1
Unrecovered	2	1	3
Total	6	6	12

Correspondence of strain-specific infection status of engorged challenge nymphs in group 3 and their test mice: There were 76 test mice that were challenged with group 3 nymphs that that had fed as larvae on multiple-strain infected mice. The group 3 nymphs can have 1 of 4 possible infection states: (1) infected with both strains, (2) infected with strain Fin-Jyv-A3 alone, (3) infected with strain NE4049 alone, and (4) uninfected. The correspondence in infection status between the engorged challenge nymphs and the test mice is shown in **Table S4**. We recovered 63 of the 76 engorged challenge nymphs (i.e., 13 engorged challenge nymphs were not recovered). Of the 63 recovered engorged nymphs, the infection status of 39 engorged nymphs was congruent with their test mice (highlighted in green in **Table S4**). There were 8 nymphs that tested negative but that transmitted one of the two strains or both strains to the test mouse (highlighted in yellow in **Table S4**). There were 10 engorged nymphs that tested positive (for either strain), but these nymphs did not transmit the infection to the mice (highlighted in blue in **Table S4**). There were 5 engorged nymphs that tested positive for a strain that was different from the strain detected in the test mice (highlighted in purple in **Table S4**). Finally, there was 1 engorged nymph that tested positive for one strain (NE4049), whereas the test mouse was infected with both strains (highlighted in red in **Table S5**).

Table S4. This contingency table shows the correspondence between the infection status of the engorged challenge nymphs (rows) from group 3 and the test mice (columns). Group 3 challenge nymphs took their larval blood meal from donor mice infected with both strains Fin-Jyv-A3 and NE4049 and therefore have four possible states: (1) infected with strain Fin-Jyv-A3 alone, (2) infected with strain NE4049 alone, (3) infected with both strains, or (4) uninfected. The row titled ‘Unrecovered’ refers to engorged nymphs that were not recovered and were therefore not tested with respect to their strain-specific infection status.

	Infection status of mice				
Infection status of engorged nymphs	Fin-Jyv-A3	NE4049	Coinfected	Uninfected	Total
Fin-Jyv-A3	14	2	0	3	19
NE4049	3	3	1	7	14
Coinfected	0	0	9	0	9
Uninfected	6	1	1	13	21
Unrecovered	3	3	0	7	13
Total	26	9	11	30	76

In summary, we recovered 81 of the 100 engorged challenge nymphs (i.e., 19 engorged challenge nymphs were not recovered). Of the 81 recovered engorged nymphs, the infection

status of 49 engorged nymphs was congruent with their test mice (highlighted in green in **Table S5**). There were 9 nymphs that tested negative but that transmitted one of the two strains or both strains to the test mouse (highlighted in yellow in **Table S5**). There were 17 engorged nymphs that tested positive (for either strain), but these nymphs did not transmit the infection to the mice (highlighted in blue in **Table S5**). There were 5 engorged nymphs that tested positive for a strain that was different from the strain detected in the test mice (highlighted in purple in **Table S5**). Finally, there was 1 engorged nymph that tested positive for one strain (NE4049), whereas the test mouse was infected with both strains (highlighted in red in **Table S5**).

Table S5. This contingency table shows the correspondence between the infection status of the 100 engorged challenge nymphs (rows) and the 100 test mice (columns). The row titled 'Unrecovered' refers to engorged nymphs that were not recovered and were therefore not tested with respect to their strain-specific infection status.

	Infection status of mice				
Infection status of engorged nymphs	Fin-Jyv-A3	NE4049	Coinfected	Uninfected	Total
Fin-Jyv-A3	20	2	0	5	27
NE4049	3	6	1	12	22
Coinfected	0	0	9	0	9
Uninfected	6	2	1	14	23
Unrecovered	5	5	0	9	19
Total	34	15	11	40	100

Section 3 – Effects of the donor mouse infection status and strain on the infection status of the unfed challenge nymphs

The purpose of this analysis was to test whether multiple-strain infection in the donor mice reduced acquisition of the two *B. afzelii* strains (Fin-Jyv-A3, NE4049) by the immature *I. ricinus* ticks that fed on these mice (i.e., larvae that fed on the donor mice molted into the unfed challenge nymphs). This analysis was restricted to the 30 donor mice that infected a high proportion of challenge nymphs with the 3 infection types of interest.

A GLMM with binomial errors was used to model the infection status of the unfed challenge nymphs. The fixed factors included donor mouse infection status (single strain, multiple strain), strain (Fin-Jyv-A3, NE4049), and their interaction; the identity of the donor mice was modelled as a random factor. Type II Wald chisquare tests were used to determine the statistical significance of the fixed factors (**Table S5**). The interaction between donor mouse infection status and strain was not significant (Δ dev = 3.675, Δ df = 1, p = 0.055) and was removed from the model. In the simplified model, donor mouse infection status was significant (Δ dev = 6.171, Δ df = 1, p = 0.013) but strain was not (Δ dev = 0.005, Δ df = 1, p = 0.945).

The parameter estimates indicated that unfed challenge nymphs that had fed as larvae on donor mice infected with both strains had a lower strain infection prevalence compared to unfed challenge nymphs that had fed as larvae on donor mice infected with a single strain (**Table S6**). The estimated marginal means (EMMs) were calculated in **Table S7** using the parameter estimates from the model in **Table S6**. Averaged across the two strains, the mean strain infection status for nymphs that fed as larvae on donor mice with single strain infections was 85.9% (95% CI = 76.9 – 91.7%) and for nymphs that fed as larvae on donor mice infected with both strains it was 66.6% (95% CI = 51.0 – 79.3%). Thus, multiple-strain infections in the donor mice reduced strain acquisition by 22.4%; 100*(85.9 - 66.6)/ 85.9 = 22.4%.

Table S5. GLMM of the infection status of the unfed challenge nymphs as a function of donor mouse infection status (single strain, multiple strain) and strain (Fin-Jyv-A3, NE4049). Shown are the change in deviance, the change in degrees of freedom, and the associated p-values.

Model	Factor	Δ Dev	Δ Df	p
Full	Donor mouse:strain	3.675	1	0.055
Simplified	Donor mouse	6.171	1	0.013
Simplified	Strain	0.005	1	0.945

Table S6. The parameter estimates on the logit scale are shown for the GLMM of the infection status of the unfed challenge nymphs in **Table S5**. The model included the fixed factors of donor mouse infection status (single strain, multiple strain) and strain (Fin-Jyv-A3, NE4049). The parameters included an intercept (defined for nymphs that fed as larvae on donor mice infected with strain Fin-Jyv-A3 only) and contrasts (expressed as the difference between the levels of a factor). Shown are the parameter estimates (Estimates), standard errors (SE), z-values, and the associated p-values (p).

Parameter	Factor	Estimates	SE	z-value	p
Intercept	Single strain & Fin-Jyv-A3	1.818	0.390	4.659	<0.00001
Contrast	Multiple strain – Single strain	-1.112	0.445	-2.498	0.013
Contrast	NE4049 – Fin-Jyv-A3	-0.029	0.442	-0.067	0.947

Table S7. The estimated marginal means (EMMs) were calculated for the four combinations of donor mouse infection status (single strain, multiple strain) and strain (Fin-Jyv-A3, NE4049) using the parameter estimates in **Table S6**. Shown are the EMMs on the logit scale (Mean 1), their standard errors (SE), the degrees of freedom (df), and the lower limit (LL1) and upper limit (UL1) of the 95% confidence interval. Also shown are the EMMs on the proportion scale (Mean 2) and the lower limit (LL1) and upper limit (UL1) of the 95% confidence interval.

Donor mouse	Strain	Mean1	SE	df	LL1	UL1	Mean2	LL2	UL2
Single strain		1.806	0.307	Inf	1.205	2.408	0.859	0.769	0.917
Multiple strain		0.692	0.333	Inf	0.039	1.344	0.666	0.510	0.793
Single strain	Fin-Jyv-A3	1.821	0.396	Inf	1.045	2.597	0.861	0.740	0.931
Multiple strain	Fin-Jyv-A3	0.707	0.403	Inf	-0.082	1.496	0.670	0.479	0.817
Single strain	NE4049	1.791	0.362	Inf	1.081	2.500	0.857	0.747	0.924
Multiple strain	NE4049	0.676	0.399	Inf	-0.105	1.458	0.663	0.474	0.811

Section 4 – Effects of strain and nymphal age on the strain abundance of the unfed challenge nymphs

The purpose of this analysis was to compare the abundance of the two strains of *B. afzelii* (Fin-Jyv-A3, NE4049) in the *I. ricinus* unfed challenge nymphs that had been frozen at either 1 month of age or 4 months after the larva-to-nymph molt. This analysis was restricted to the 279 unfed challenge nymphs that had fed as larvae on the 18 donor mice that were infected with strain Fin-Jyv-A3 alone or strain NE4049 alone (i.e., unfed challenge nymphs that had fed as larvae on the 12 donor mice infected with both strains were excluded). The abundance of each strain in the unfed challenge nymphs was estimated by the number of *flagellin* gene copies.

An LMM was used to model the log10-transformed strain abundance in the unfed challenge nymphs. The fixed factors included strain (Fin-Jyv-A3, NE4049), nymphal age (1 month, 4 months) and their interaction; the identity of the donor mice was modelled as a random factor. Type II Wald chisquare tests were used to determine the statistical significance of the fixed factors (**Table S8**). The interaction between strain and nymphal age was not significant (Δ dev = 0.262, Δ df = 1, p = 0.608) and was removed from the model. In the simplified model, strain (Δ dev = 5.754, Δ df = 1, p = 0.016) and nymphal age (Δ dev = 7.366, Δ df = 1, p = 0.007) both had significant effects on the nymphal strain abundance.

The parameter estimates indicated that strain Fin-Jyv-A3 established a higher abundance in the unfed challenge nymphs compared to strain NE4049 and that 1-month-old nymphs had higher strain abundance compared to 4-month-old nymphs (**Table S9**). The estimated marginal

means (EMMs) were calculated in **Table S10** using the parameter estimates from the model in **Table S9**.

The nymphal strain abundance has units of the number of spirochetes per nymph. Averaged across the two nymphal ages, the mean nymphal strain abundance for strain Fin-Jyv-A3 (mean = 5321.4; 95% CI = 3490.8 - 8111.8) was 1.9x higher compared to strain NE4049 (mean = 2766.2; 95% CI = 1850.6 - 4134.8). Averaged across the two nymphal ages, the mean nymphal strain abundance for 1-month-old nymphs (mean = 4914.9; 95% CI = 3538.1 – 6827.5) was 1.6x higher compared to 4-month-old nymphs (mean = 2994.9; 95% CI = 2122.6 – 4225.6).

Table S8. LMM of the strain abundance of the unfed challenge nymphs as a function of strain (Fin-Jyv-A3, NE4049) and nymphal age (1-month-old, 4-month-old). Shown are the change in deviance, the change in degrees of freedom, and the associated p-values.

Model	Factor	Δ Dev	Δ Df	p
Full	Strain:Nymphal age	0.262	1	0.608
Simplified	Strain	5.754	1	0.016
Simplified	Nymphal age	7.366	1	0.007

Table S9. The parameter estimates on the log10 scale are shown for the LMM of strain abundance of the unfed challenge nymphs in **Table S8**. The model included the fixed factors of strain (Fin-Jyv-A3, NE4049) and nymphal age (1-month-old, 4-month-old). The parameters included an intercept (defined for 1-month-old nymphs that fed as larvae on donor mice infected with strain Fin-Jyv-A3 only) and contrasts (expressed as the difference between the levels of a factor). Shown are the parameter estimates (Estimates), standard errors (SE), z-values, and the associated p-values (p).

Parameter	Factor	Estimates	SE	df	t-value	p
Intercept	Fin-Jyv-A3 & 1-month-old	3.834	0.092	21.565	41.838	< 2e-16
Contrast	NE4049 – Fin-Jyv-A3	-0.284	0.118	14.495	-2.399	0.030
Contrast	4 months – 1 month	-0.215	0.079	273.115	-2.714	0.007

Table S10. The estimated marginal means were calculated for each strain (Fin-Jyv-A3, NE4049), each nymphal age (1-month-old, 4-month-old), and for the four combinations of strain and nymphal age using the parameter estimates in **Table S9**. Shown are the EMMs on the log10 scale (Mean 1), their standard errors (SE), the degrees of freedom (df), and the lower limit (LL1) and upper limit (UL1) of the 95% confidence interval. Also shown are the EMMs on strain abundance scale (Mean 2; units are number of *flagellin* copies per unfed nymph) and the lower limit (LL1) and upper limit (UL1) of the 95% confidence interval.

Strain	Nymphal age	Mean1	SE	df	LL1	UL1	Mean2	LL2	UL2
Fin-Jyv-A3		3.726	0.086	16.238	3.543	3.909	5321.4	3490.8	8111.8
NE4049		3.442	0.081	14.134	3.267	3.616	2766.2	1850.6	4134.8
	1-month-old	3.692	0.070	31.766	3.549	3.834	4914.9	3538.1	6827.5
	4-month-old	3.476	0.073	27.460	3.327	3.626	2994.9	2122.6	4225.6
Fin-Jyv-A3	1-month-old	3.834	0.092	22.671	3.643	4.024	6816.9	4400.4	10560.5
Fin-Jyv-A3	4-month-old	3.549	0.092	22.733	3.359	3.740	3543.6	2285.3	5494.6
NE4049	1-month-old	3.618	0.099	23.321	3.415	3.822	4153.9	2598.2	6641.0
NE4049	4-month-old	3.334	0.089	19.124	3.147	3.521	2159.3	1403.4	3322.2

Section 5 – GLMM to simultaneously test the effects of strain and donor mouse infection status on test mouse infection status

The purpose of this analysis was to investigate whether *B. afzelii* strain (Fin-Jyv-A3, NE4049) and the putative infection status of the challenge nymphs (infected with single strain, infected with both strains) influenced the infection status of the test mice. This analysis included all 100 test mice. The putative infection status of the challenge nymph was inferred from the infection status of the donor mouse from which it had taken its larval blood meal. This analysis includes challenge nymphs that were not infected with the focal strain, and it therefore underestimates the true probability of nymph-to-host transmission. This analysis is important because it shows that the negative effects of multiple-strain infection in the donor mice on strain acquisition by unfed challenge nymphs (see ESM Section 3) reduces the percentage of test mice that become infected following exposure to these challenge nymphs.

A GLMM with binomial errors was used to analyze the infection status of the 100 test mice as a function of donor mouse infection status (single strain, multiple strain), strain (Fin-Jyv-A3, NE4049), and their interaction; the identity of the donor mice was modelled as a random factor. Stepwise model simplification was used to remove non-significant terms. Type II Wald chisquare tests were used to determine the statistical significance of the fixed factors (**Table S11**). The interaction between focal strain and donor mouse infection status ($\chi^2 = 0.098$, df = 1, p = 0.753) was not significant and was therefore removed from the model. The infection status of the donor mouse (Δ dev = 4.684, df = 1, p = 0.030) and strain (Δ dev = 3.883, df = 1, p = 0.049) were both significant.

The parameter estimates indicated that nymphs from the multiple strain group (i.e., fed as larvae on donor mice infected with both strains) infected fewer test mice compared to nymphs from the single strain group (i.e., fed as larvae on donor mice infected with a single strain) (**Table S12**). The parameter estimates also showed that nymphs infected with strain Fin-Jyv-A3 infected a higher percentage of test mice than nymphs infected with strain NE4049 (**Table S12**). The estimated marginal means (EMMs) were calculated in **Table S13** using the parameter estimates from the model in **Table S12**.

Nymphs from the single strain group (i.e., fed as larvae on donor mice infected with a single strain) infected 58.8% of the test mice, whereas nymphs from the multiple strain group (i.e., fed as larvae on donor mice infected with both strains) infected 30.7% of the test mice (**Table S13**). Thus, the percentage of test mice that became infected was reduced by 47.8% when they were exposed to nymphs from the multiple strain group versus nymphs from the single strain group; 100*(58.8-30.7)/58.8 = 47.8%. We had previously calculated that multiple-strain infection in the donor mice reduced by 22.4% strain acquisition, which was measured in the 1-month-old and 4-month-old nymphs via qPCR. Thus, the 22.4% reduction in strain acquisition by the unfed challenge nymphs resulted in a 47.8% reduction in infection success when those nymphs were fed on the test mice. The challenge nymphs were 10 months old when they were fed upon the test mice. Thus, one possible explanation for the discrepancy between the 22.4% reduction in strain acquisition by the unfed challenge nymphs and the 47.8% reduction in infection success of the test mice is that the spirochete populations inside the challenge nymphs became less viable as the nymphs aged from 4 months to 10 months. Nymphs in experiment 1 infected 55.7% of the test mice with strain Fin-Jyv-A3, whereas nymphs in experiment 2 infected

33.4% of the test mice with strain NE4049 (**Table S13**). As mentioned previously, these analyses include uninfected challenge nymphs, and they therefore underestimate the true probability of nymph-to-host transmission.

Table S11. GLMM of the infection status of the test mice as a function of donor mouse infection status (single strain, multiple strain) and strain (Fin-Jyv-A3, NE4049). Shown are the change in deviance, the change in degrees of freedom, and the associated p-values.

Model	Factor	Δ Dev	Δ Df	p
Full	Donor mouse:strain	0.098	1	0.753
Simplified	Donor mouse	4.684	1	0.030
Simplified	Strain	3.883	1	0.049

Table S12. The parameter estimates on the logit scale are shown for the GLMM of the infection status of the test mice in **Table S11**. The model included the fixed factors of donor mouse infection status (single strain, multiple strain) and strain (Fin-Jyv-A3, NE4049). The parameters include an intercept (defined for test mice that were fed upon by nymphs that fed as larvae on donor mice infected with strain Fin-Jyv-A3 only) and contrasts (expressed as the difference between the levels of a factor). Shown are the parameter estimates (Estimates), standard errors (SE), z-values, and the associated p-values (p).

Parameter	Factor	Estimates	SE	z-value	p
Intercept	Single strain & Fin-Jyv-A3	0.815	0.507	1.607	0.108
Contrast	Multiple – Single	-1.168	0.540	-2.164	0.031
Contrast	NE4049 – Fin-Jyv-A3	-0.921	0.467	-1.971	0.049

Table S13. The estimated marginal means (EMMs) were calculated for each donor mouse infection status (single strain, multiple strain), each strain (Fin-Jyv-A3, NE4049), and for the four combinations of donor mouse infection status and strain using the parameter estimates in **Table S12**. Shown are the EMMs on the logit scale (Mean 1), their standard errors (SE), the degrees of freedom (df), and the lower limit (LL1) and upper limit (UL1) of the 95% confidence interval. Also shown are the EMMs on the proportion scale (Mean 2) and the lower limit (LL1) and upper limit (UL1) of the 95% confidence interval.

Donor mouse	Strain	Mean1	SE	df	LL1	UL1	Mean2	LL2	UL2
Single strain		0.355	0.430	Inf	-0.488	1.198	0.588	0.380	0.768
Multiple strain		-0.813	0.294	Inf	-1.390	-0.237	0.307	0.199	0.441
	Fin-Jyv-A3	0.231	0.326	Inf	-0.408	0.871	0.557	0.399	0.705
	NE4049	-0.690	0.359	Inf	-1.393	0.013	0.334	0.199	0.503
Single strain	Fin-Jyv-A3	0.815	0.507	Inf	-0.179	1.809	0.693	0.455	0.859
Multiple strain	Fin-Jyv-A3	-0.353	0.318	Inf	-0.977	0.271	0.413	0.273	0.567
Single strain	NE4049	-0.106	0.471	Inf	-1.029	0.818	0.474	0.263	0.694

Multiple strain	NE4049	-1.274	0.425	Inf	-2.108	-0.440	0.219	0.108	0.392

Section 6 – Proportion tests to separately compare the effects of donor mouse infection status and strain on the infection prevalence of the test mice

We used proportion tests to determine the effects of multiple strain infection and strain on the percentage of test mice that became infected after the infestation with the challenge nymphs. After combining experiments 1 and 2, the strain infection prevalence of the test mice infested with the nymphs from the single strain treatment (58.3% = 14/24) was 1.8x higher compared to the test mice infested with the nymphs from the multiple strain treatment (31.6% = 24/76), and this difference was significant (Proportion test: $\chi^2 = 4.464$, df = 1, p = 0.035; **Table S14**). After combining experiments 1 and 2, the percentage of test mice infected with strain Fin-Jyv-A3 (48.0% = 24/50) was 1.7x higher compared to strain NE4049 (28.0% = 14/50), and this difference was almost significant ($\chi^2 = 3.438$, df = 1, p = 0.064; **Table S14**). The results from this proportion test analysis are very similar to the previous GLMM analysis.

Table S14. Estimates of the percentage of test mice infected with *B. afzelii* following exposure to putatively infected *I. ricinus* nymphs are shown for the two donor mouse infection treatments (single strain versus multiple strain), the two strains (Fin-Jyv-A3 versus NE4049), and the four combinations of these two factors. The percentage of infected test mice is an underestimate of the probability of nymph-to-host transmission because some test mice were challenged with nymphs that were not infected with the focal strain. The number of infected test mice divided by the total number of test mice and the percentage of infected test mice are shown. The outcome of the proportion tests are shown for the pairwise comparisons including the Chi-square statistic (χ^2), degrees of freedom (df), and p-value (p). As expected, the parameter estimates from the GLMM in Table S13 are very similar to the percentage of infected test mice in Table S14.

Donor mouse	Strain	Infected/Total	% Infected	χ^2	df	p
Single strain	NA	14/24	58.3%	4.464	1	0.035
Multiple strain	NA	24/76	31.6%			
NA	Fin-Jyv-A3	24/50	48.0%	3.438	1	0.064
NA	NE4049	14/50	28.0%			
Single strain	Fin-Jyv-A3	8/12	66.7%	1.33	1	0.249
Multiple strain	Fin-Jyv-A3	16/38	42.1%			
Single strain	NE4049	6/12	50.0%	2.49	1	0.115
Multiple strain	NE4049	8/38	21.1%			

Section 7 – Infection status of the engorged challenge nymph is the best predictor of the infection status of the test mice

We had estimates that the challenge nymph contained the focal strain and the competitor strains both before and after the blood meal on the test mice. The estimate that the 10-month-old challenge nymph contained the focal strain before the blood meal was based on the strain-specific qPCR-based testing of the 1-month-old and 4-month-old nymphs that had fed as larvae on each donor mouse (Table S1). The estimate that the 10-month-old challenge nymph contained the focal strain after the blood meal was based on strain-specific qPCR-based testing of the engorged nymph. For convenience, we will refer to these two variables as pre-infestation estimates versus post-infestation estimates of the infection status of the challenge nymph.

This analysis was restricted to the subset of 81 test mice for which we recovered and tested the engorged challenge nymph. Each of the 81 test mice can be used to estimate the probability of nymph-to-host transmission for strain Fin-Jyv-A3 and for strain NE4049. For this reason, each of the 81 test mice appeared twice in the analysis: once for focal strain Fin-Jyv-A3 and competitor strain NE4049 and once for focal strain NE4049 and competitor strain Fin-Jyv-A3. Thus, the total sample size was 81 x 2 = 162 test mice.

We used AIC-based model selection to determine whether the pre-infestation estimates versus the post-infestation estimates (of the infection status of the challenge nymph) explained more of the variation in the infection status of the test mice. The models that included the infection status of the engorged challenge nymph (post-infestation estimates) had 100.0% of the support, whereas models that included the probability of infection for the unfed challenge nymphs (pre-infestation estimates) had 0.0% of the support (**Table S15**). Thus, models with post-infestation estimates of the infection status of the challenge nymph were better compared to models with pre-infestation estimates. The best model has the fewest number of parameters and is within 1 AICc unit of the top model. By this definition, model 8, which contains the effects of strain and the focal strain infection status of the engorged nymph, was the best model (**Table S15**). Starting with the full model (model 6 in **Table S15**), we determined whether stepwise model simplification would converge on the same best model (see section 8).

Table S15. AIC-based model selection table is shown for 10 models that analyzed the infection status of 81 test mice for which an engorged nymph was recovered. The explanatory variables included strain, focal strain infection status of the engorged nymph (Eng.foc), competitor strain infection status of the engorged nymph (Eng.comp), probability that the unfed challenge nymph was putatively infected with the focal strain (P(Unfed.foc)), and probability that the unfed challenge nymph was putatively infected with the competitor strain (P(Unfed.comp)). For each model, the following are shown: the model structure, the degrees of freedom for the parameters (df), loglikelihood (logLik), corrected Akaike information criterion (AICc), the change in the AICc value from the top model, and the weight of the model.

Model ID	Model structure	df	logLik	AICc	Δ AICc	weight
7	Y ~ Strain + Eng.foc + Eng.comp	5	-79.126	168.636	0.000	0.482
8	Y ~ Strain + Eng.foc	4	-80.550	169.354	0.719	0.337
6	Y ~ Strain + Eng.foc + Eng.comp + Strain:Eng.foc	6	-79.063	170.668	2.032	0.174
9	Y ~ Eng.foc	3	-85.482	177.116	8.481	0.007
3	Y ~ Strain + P(Unfed.foc)	4	-95.040	198.334	29.698	0.000
2	Y ~ Strain + P(Unfed.foc) + P(Unfed.comp)	5	-94.865	200.115	31.480	0.000
1	Y ~ Strain + P(Unfed.foc) + P(Unfed.comp) + Strain:P(Unfed.foc)	6	-94.857	202.255	33.620	0.000
4	Y ~ P(Unfed.foc)	3	-102.241	210.633	41.998	0.000
5	Y ~ Strain	3	-102.465	211.082	42.446	0.000
10	Y ~ 1	2	-107.300	218.676	50.040	0.000

Section 8 – Effect of strain and infection status of the engorged challenge nymph on test mouse infection status

The purpose of this analysis was to determine whether multiple strain infection in the engorged challenge nymph and *B. afzelii* strain (Fin-Jyv-A3 and NE4049) influenced the probability of nymph-to-host transmission of the strains to the test mice. This analysis was restricted to the 81 test mice for which we had recovered the engorged challenge nymph and determined its strain-specific infection status using PCR-based methods. In this analysis, we use the infection status of the engorged nymph with respect to the focal strain and competitor strain as two explanatory factors. The importance of this analysis is that it provides the best estimates of the probability of nymph-to-host transmission for each strain of *B. afzelii*.

Starting with model 6 in **Table S15**, we determined whether stepwise model simplification converged on the same best model (model 8 in **Table S15**). Model 6 was a GLMM with binomial errors that analyzed test mouse infection status as a function of strain, focal strain infection status of the engorged nymph, competitor strain infection status of the engorged nymph, and the 2-way interaction between focal strain infection status of the engorged nymph and competitor strain infection status of the engorged nymph. Type II Wald chisquare tests were used to determine the statistical significance of the fixed factors (**Table S16**).

In model 6, the interaction between focal strain infection status of the engorged nymph and competitor strain infection status of the engorged nymph was not significant (Δ dev = 0.125, df = 1, p = 0.723) and was removed from the model. In model 7, competitor strain infection status of the engorged nymph was not significant (Δ dev = 2.716, df = 1, p = 0.099). In model 8, strain (Δ dev = 9.180, df = 1, p = 0.002) and focal strain infection status of the engorged nymph (Δ dev = 36.254, df = 1, p = 1.732e-09) were both significant.

Engorged challenge nymphs that tested negative for *B. afzelii* via qPCR still transmitted *B. afzelii* to the test mice, but at a much lower probability than engorged challenge nymphs that tested positive for *B. afzelii* (**Tables S17 and S18**). Averaged across the two strains, nymph-to-host transmission for infected engorged nymphs (mean = 67.6%; 95% CI = 55.0 – 78.0%) was 4.3x higher compared to uninfected engorged nymphs (mean = 15.6%; 95% CI = 9.5 – 24.6%). For strain Fin-Jyv-A3, nymph-to-host transmission for infected engorged nymphs (mean = 79.3%; 95% CI = 65.7 – 88.5%) was 3.1x higher compared to uninfected engorged nymphs (mean = 25.4%; 95% CI = 15.7 – 38.5%). For strain NE4049, nymph-to-host transmission for infected engorged nymphs (mean = 53.0%; 95% CI = 37.5 – 68.0%) was 5.8x higher compared to uninfected engorged nymphs (mean = 9.1%; 95% CI = 4.5 – 17.8%).

Nymph-to-host transmission was higher for strain Fin-Jyv-A3 compared to strain NE4049 (**Tables S17 and S18**). If the engorged challenge nymph was infected with the focal strain, nymph-to-host transmission of strain Fin-Jyv-A3 (mean = 79.3%; 95% CI = 65.7 – 88.5%) was 1.5x higher compared to strain NE4049 (mean = 53.0%; 95% CI = 37.5 – 68.0%). For engorged challenge nymphs that tested negative for the focal strain, nymph-to-host transmission of strain Fin-Jyv-A3 (mean = 25.4%; 95% CI = 15.7 – 38.5%) was 2.8x higher compared to strain NE4049 (mean = 9.1%; 95% CI = 4.5 – 17.8%).

Table S16. GLMM of the infection status of the test mice as a function of strain (Fin-Jyv-A3, NE4049), focal strain infection status of the engorged nymph (FSIS_EN; uninfected, infected), and competitor strain infection status of the engorged nymph (CSIS_EN; absent, present). Shown are the change in deviance (Δ Dev), the change in degrees of freedom (Δ Df), and the associated p-values (p).

Model	Factor	Δ Dev	Δ Df	p
6	Strain	9.762	1	0.002
6	Focal strain infection status	35.981	1	< 0.001
6	Competitor strain infection status	2.715	1	0.099
6	FSIS_EN:CSIS_EN	0.125	1	0.723
7	Strain	9.729	1	0.002
7	Focal strain infection status	35.985	1	< 0.001
7	Competitor strain infection status	2.716	1	0.099
8	Strain	9.180	1	0.002
8	Focal strain infection status	36.254	1	< 0.001

Table S17. The parameter estimates on the logit scale are shown for the GLMM of the infection status of the test mice (models 7 and 8 in **Table S16**). The model included the fixed factors of strain (Fin-Jyv-A3, NE4049), focal strain infection status of the engorged nymph (FSIS_EN; uninfected, infected), and competitor strain infection status of the engorged nymph (CSIS_EN; absent, present). The parameters include an intercept (defined for test mice that were fed upon by nymph uninfected with strain Fin-Jyv-A3 and no competitor strain present) and contrasts (expressed as the difference between the levels of a factor). Shown are the parameter estimates (Estimates), standard errors (SE), z-values, and the associated p-values (p).

Model	Parameter	Factor	Estimates	SE	z-value	p
7	Intercept	Reference 7*	-1.461	0.403	-3.625	< 0.001
7	Contrast	NE4049 – Fin-Jyv-A3	-1.277	0.409	-3.119	0.002
7	Contrast	Focal positive – Focal negative	2.655	0.443	5.999	< 0.001
7	Contrast	Comp positive – Comp negative	0.720	0.437	1.648	0.099
8	Intercept	Reference 8*	-1.076	0.310	-3.467	< 0.001
8	Contrast	NE4049 – Fin-Jyv-A3	-1.223	0.404	-3.030	0.002
8	Contrast	Focal positive – Focal negative	2.421	0.402	6.021	< 0.001

* Reference 7 = Fin-Jyv-A3 is the focal strain, the engorged nymph tests negative for the focal strain, and the engorged nymph tests negative for the competitor strain

* Reference 8 = Fin-Jyv-A3 is the focal strain, and the engorged nymph tests negative for the focal strain

Table S18. The estimated marginal means (EMMs) were calculated for each strain (Fin-Jyv-A3, NE4049), each focal strain infection status of the engorged nymph (FSIS_EN; uninfected, infected) and for the four combinations of strain and FSIS_EN using the parameter estimates in **Table S17**. Shown are the EMMs on the logit scale (Mean 1), their standard errors (SE), the degrees of freedom (df), and the lower limit (LL1) and upper limit (UL1) of the 95% confidence interval. Also shown are the EMMs on the proportion scale (Mean 2) and the lower limit (LL1) and upper limit (UL1) of the 95% confidence interval.

Strain	FSIS_EN	Mean1	SE	df	LL1	UL1	Mean2	LL2	UL2
Fin-Jyv-A3		0.134	0.266	Inf	-0.386	0.655	0.534	0.405	0.658
NE4049		-1.089	0.297	Inf	-1.670	-0.508	0.252	0.158	0.376
	Uninfected	-1.688	0.289	Inf	-2.255	-1.120	0.156	0.095	0.246
	Infected	0.733	0.272	Inf	0.200	1.267	0.676	0.550	0.780
Fin-Jyv-A3	Uninfected	-1.076	0.310	Inf	-1.684	-0.468	0.254	0.157	0.385
Fin-Jyv-A3	Infected	1.345	0.355	Inf	0.650	2.040	0.793	0.657	0.885
NE4049	Uninfected	-2.299	0.391	Inf	-3.065	-1.533	0.091	0.045	0.178
NE4049	Infected	0.122	0.322	Inf	-0.510	0.754	0.530	0.375	0.680

Section 9 – Literature review of estimates of nymph-to-host transmission of *B. burgdorferi* sl pathogens

Methods for literature review: We searched the literature for studies that had estimates of nymph-to-host transmission of *B. burgdorferi* sl pathogens. For each study, we recorded the *B. burgdorferi* sl species, *Ixodes* tick species, vertebrate host species, number of nymphs with which each host was infested (often indicated as a range), nymphal infection prevalence (NIP; proportion of infected nymphs), number of infected nymphs per host (often indicated as a range), time point at which the nymphs were removed from the host, and the host infection prevalence (HIP; proportion of hosts that became infected following nymphal challenge). As our focus was on the probability of nymph-to-host transmission for an uninterrupted nymphal blood meal, only those nymphs allowed to feed ≥ 72 hours were included in the study.

Results of literature review: We found 18 studies that had investigated nymph-to-host transmission for *B. burgdorferi* sl, which we subdivided into 30 estimates of nymph-to-host transmission (i.e., some studies provided multiple estimates for different strains, different numbers of infected nymphs, and/or different time points). *Borrelia burgdorferi* sl pathogens included: *B. afzelii*, *B. burgdorferi* ss, *B. bissettii*, *B. mayonii*, and the relapsing fever spirochete *B. miyamotoi* (**Table S19**). *Ixodes* tick species included *I. ricinus, I. pacificus, I. scapularis*, and *I. spinipalpis* (**Table S19**). Vertebrate hosts included *Mesocricetus auratus*, *Mus musculus*, *Myodes glareolus*, *Peromyscus leucopus*, and *Peromyscus maniculatus* (**Table S19**). Many of these studies had been conducted to determine how the probability of nymph-to-host transmission of *B. burgdorferi* sl increases over the duration of nymphal attachment (3-8). For many studies, the hosts had been infected with multiple infected nymphs, and the proportion of hosts infected following the nymphal challenge (HIP = host infection prevalence) is therefore an overestimate of the probability of nymph-to-host transmission

(**Table S19**). Few studies infested hosts with single infected nymphs, which is the best experimental design for estimating nymph-to-host transmission (3, 6, 7, 9-11). One study on *B. mayonii* demonstrates the importance of the number of infected nymphs on the HIP; the HIP was 57.1% versus 83.3% for CD-1 mice that had been infested with 1 versus 2 infected *I. scapularis* nymphs (6). Very few studies indicated the age of the nymphs when they were fed on the hosts (**Table S19**).

Table S19. Estimates of nymph-to-host transmission of different *Borrelia burgdorferi* sensu lato species and the relapsing fever spirochete *Borrelia miyamotoi*. Shown for each study are the *Borrelia* species and strain, *Ixodes* tick species, vertebrate host species and strain, nymphs per host, nymphal infection prevalence (NIP), number of infected nymphs per host, time point to which nymphs were allowed to feed, nymph age, host infection prevalence (HIP), and the reference. For studies that exposed hosts to a single infected nymph, the HIP is an estimate of the probability of nymph-to-host transmission.

Borrelia Species [a]; Strain	Tick Species [b]	Host Species [c]; Strain	Nymphs Per host	NIP (%)	Infected Nymphs Per host	Time point	Nymph age (months)	HIP (%)	Reference
Bafz; NE496, NE2963	IR	MM; AKR/N	6	60-80%	3.6-4.8 [d]	≥ 72 h	NA	77.8% (7/9)	(8)
Bafz	IR	MM; BALB/c	3-20	15.4-100%	3	Repletion	NA	82.2% (37/45)	(12)
Bafz; Fin-Jyv-A3	IR	MM; BALB/c	1	63.6-100.0%	1	Repletion	10-11	79.3% (64/81)	Present
Bafz; NE4049	IR	MM; BALB/c	1	53.3-100.0%	1	Repletion	10-11	53.0% (43/81)	Present
Bafz; NE4049	IR	MM; BALB/c ByJ	2-9	14.3-50.0	1-1.5	Repletion	1	94.4% (17/18)	(9)
Bafz; NE4049	IR	MM; BALB/c ByJ	1-2	50.0-100.0	1	Repletion	1	100% (10/10)	(9)
Bafz; NE4049, E61	IR	MG	3	91.3%	1-3	Repletion	NA	89.5% (34/38)	(13)
Bafz; Fin-Jyv-A3, NE4049	IR	MG	3	96.8%	1-3	Repletion	NA	100.0% (50/50)	(13)
Bafz; NE4049	IR	MG	4	77.9%	1-4	Repletion	NA	100.0% (8/8)	(14)
Bafz; Fin-Jyv-A3	IR	MG	4	91.8%	1-4	Repletion	NA	95.2% (20/21)	(14)
Bbss; ZS7, NE1849	IR	MM; AKR/N	6	60-70%	3.6-4.2 [d]	≥ 72 h	NA	44.4% (4/9)	(8)
Bbss; JD1	IS	MM	1	NA	1	72 h	NA	56.2% (9/16)	(3)
Bbss; B31	IS	MM	1	NA	1	72 h	NA	70.6% (12/17)	(3)
Bbss; Field strain	IS	MM	1	30.0%	1	96 h	NA	93.8% (15/16)	(3)
Bbss; JD1	ID	MA	3	90.0-100.0%	1-3	≥ 72 h	NA	83.3% (5/6)	(4)
Bbss; JD1	ID	PL	3	90.0-100.0%	1-3	≥ 72 h	NA	100.0% (8/8)	(4)
Bbss; CA4	IP	PM	15-20	20.0-60.0%	1-2	≥ 96 h	NA	80% (8/10)	(5)
Bbss; JD1	ID	MA	NA	83.0%	1-14	Repletion	NA	100.0% (4/4)	(15)
Bbss; B31	IS	MM; CD-1	5	NA	1-4	Repletion	2	88.9% (16/18)	(16)
Bbss; B31, JD1	IS	MM; CD-1	3-12	75.0-100.0%	3-12	Repletion	12	100% (3/3)	(17)

Bbis	ISP	PM	3-22	35-80%	2-12	Repletion	NA	100.0% (8/8)	(18)
Bbis	IP	PM	1-7	50-75%	1-6	Repletion	NA	76.5% (13/17)	(18)
Bmayo; MN14-1420	IS	MM; CD-1	2	25-50%	1	72 h	NA	31.2% (5/16)	(6)
Bmayo; MN14-1420	IS	MM; CD-1	2	25-50%	2	72 h	NA	71.4% (5/7)	(6)
Bmayo; MN14-1420	IS	MM; CD-1	2	25-50%	1	Repletion	NA	57.1% (8/14)	(6)
Bmayo; MN14-1420	IS	MM; CD-1	2	25-50%	2	Repletion	NA	83.3% (5/6)	(6)
Bmayo; MN14-1420	IS	MM; CD-1	1-10	1.3-47.5%	1	Repletion	NA	40.0% (6/15)	(10)
Bmayo; MN-17-4755	IS	PL	20	NA	1 to 10	Repletion	NA	91.3% (21/23)	(19)
Bmiya	IS	MM; CD-1	1-2	62-91%	1-2	Repletion	larvae	62.1% (18/29)	(11)
Bmiya	IS	MM; CD-1	1	97%	1	Repletion	NA	73.3% (22/30)	(7)

[a] Borrelia species: Bafz = *Borrelia afzelii*; Bbis = *Borrelia bissettii*; Bbss = *Borrelia burgdorferi* sensu stricto; Bmayo = *Borrelia mayonii*; Bmiya = *Borrelia miyamotoi*

[b] Tick species: ID = *Ixodes dammini*; IP = *Ixodes pacificus*; IR = *Ixodes ricinus*; IS = *Ixodes scapularis*; ISP = *Ixodes spinipalpis*

[c] Host species: *Mesocricetus auratus* = MA; MM = *Mus musculus*; MG = *Myodes glareolus*; PL = *Peromyscus leucopus*; PM = Peromyscus maniculatus

[d] This study did not test the engorged nymphs to determine whether they were infected. The number of infected nymphs per host was estimated by multiplying the number of nymphs per host by the NIP.

References

1. Genné D, Sarr A, Gomez-Chamorro A, Durand J, Cayol C, Rais O, Voordouw MJ. 2018. Competition between strains of *Borrelia afzelii* inside the rodent host and the tick vector. P Roy Soc B-Biol Sci 285.
2. Genné D, Sarr A, Rais O, Voordouw MJ. 2019. Competition between strains of *Borrelia afzelii* in immature *Ixodes ricinus* ticks is not affected by season. Front Cell Infect Microbiol 9:1-14.
3. des Vignes F, Piesman J, Heffernan R, Schulze TL, Stafford KC, Fish D. 2001. Effect of tick removal on transmission of *Borrelia burgdorferi* and *Ehrlichia phagocytophila* by *Ixodes scapularis* nymphs. J Infect Dis 183:773-778.
4. Piesman J, Mather TN, Sinsky RJ, Spielman A. 1987. Duration of tick attachment and *Borrelia burgdorferi* transmission. J Clin Microbiol 25:557-558.
5. Peavey CA, Lane RS. 1995. Transmission of *Borrelia burgdorferi* by *Ixodes pacificus* nymphs and reservoir competence of deer mice (*Peromyscus maniculatus*) infected by tick-bite. J Parasitol 81:175-178.
6. Dolan MC, Breuner NE, Hojgaard A, Boegler KA, Hoxmeier JC, Replogle AJ, Eisen L. 2017. Transmission of the Lyme disease spirochete *Borrelia mayonii* in relation to duration of attachment by nymphal *Ixodes scapularis* (Acari: Ixodidae). J Med Entomol 54:1360-1364.
7. Breuner NE, Dolan MC, Replogle AJ, Sexton C, Hojgaard A, Boegler KA, Clark RJ, Eisen L. 2017. Transmission of *Borrelia miyamotoi* sensu lato relapsing fever group spirochetes in relation to duration of attachment by *Ixodes scapularis* nymphs. Ticks Tick Borne Dis 8:677-681.
8. Crippa M, Rais O, Gern L. 2002. Investigations on the mode and dynamics of transmission and infectivity of *Borrelia burgdorferi* sensu stricto and *Borrelia afzelii* in *Ixodes ricinus* ticks. Vector Borne and Zoonotic Diseases 2:3-9.
9. Belli A, Sarr A, Rais O, Rego ROM, Voordouw MJ. 2017. Ticks infected via co-feeding transmission can transmit Lyme borreliosis to vertebrate hosts. Scientific Reports 7.
10. Eisen L, Breuner NE, Hojgaard A, Hoxmeier JC, Pilgard MA, Replogle AJ, Biggerstaff BJ, Dolan MC. 2017. Comparison of vector efficiency of *Ixodes scapularis* (Acari: Ixodidae) from the Northeast and Upper Midwest of the United States for the Lyme disease spirochete *Borrelia mayonii*. J Med Entomol 54:239-242.
11. Breuner NE, Hojgaard A, Replogle AJ, Boegler KA, Eisen L. 2018. Transmission of the relapsing fever spirochete, *Borrelia miyamotoi*, by single transovarially-infected larval *Ixodes scapularis* ticks. Ticks Tick Borne Dis 9:1464-1467.
12. Tonetti N, Voordouw MJ, Durand J, Monnier S, Gern L. 2015. Genetic variation in transmission success of the Lyme borreliosis pathogen *Borrelia afzelii*. Ticks Tick Borne Dis 6:334-343.
13. Gomez-Chamorro A, Battilotti F, Cayol C, Mappes T, Koskela E, Boulanger N, Genné D, Sarr A, Voordouw MJ. 2019. Susceptibility to infection with *Borrelia afzelii* and TLR2 polymorphism in a wild reservoir host. Scientific Reports 9.
14. Gomez-Chamorro A, Heinrich V, Sarr A, Roethlisberger O, Genné D, Bregnard C, Jacquet M, Voordouw MJ. 2019. Maternal antibodies provide bank voles with strain-specific protection against infection by the Lyme disease pathogen. Appl Environ Microbiol 85.

15. Piesman J, Sinsky RJ. 1988. Ability of *Ixodes scapularis*, *Dermacentor variabilis*, and *Amblyomma americanum* (Acari, Ixodidae) to acquire, maintain, and transmit Lyme disease spirochetes (*Borrelia burgdorferi*). J Med Entomol 25:336-339.
16. Goddard J, Embers M, Hojgaard A, Piesman J. 2015. Comparison of tick feeding success and vector competence for *Borrelia burgdorferi* among immature *Ixodes scapularis* (Ixodida: Ixodidae) of both southern and northern clades. J Med Entomol 52:81-85.
17. Jacobs MB, Purcell JE, Philipp MT. 2003. *Ixodes scapularis* ticks (Acari: Ixodidae) from Louisiana are competent to transmit *Borrelia burgdorferi*, the agent of Lyme borreliosis. J Med Entomol 40:964-967.
18. Eisen L, Dolan MC, Piesman J, Lane RS. 2003. Vector competence of *Ixodes pacificus* and *I. spinipalpis* (Acari: Ixodidae), and reservoir competence of the dusky-footed woodrat (*Neotoma fuscipes*) and the deer mouse (*Peromyscus maniculatus*), for *Borrelia bissettii*. J Med Entomol 40:311-320.
19. Parise CM, Breuner NE, Hojgaard A, Osikowicz LM, Replogle AJ, Eisen RJ, Eisen L. 2020. Experimental demonstration of reservoir competence of the white-footed mouse, *Peromyscus leucopus* (Rodentia: Cricetidae), for the Lyme disease spirochete, *Borrelia mayonii* (Spirochaetales: Spirochaetaceae). J Med Entomol 57:927-932.
